# Supplementary material for: Transfer of motor learning is associated with patterns of activity in the default mode network
Source: PLoS Biol. 2025 Aug 14;23(8):e3003268. doi: 10.1371/journal.pbio.3003268 (PMC12352662; doi:10.1371/journal.pbio.3003268)
Supplement: S1 Text — Fig A in S1 Text: Comparison of effector- and learning-related changes in manifold eccentricity using different numbers of principal components (PCs). Fig B: Functional connectivity properties that underlie manifold eccentricity. Fig C: Brain regions showing a Task Epoch × Hand interaction effect. Fig D: Effector- and learning/transfer-related changes in connectivity do not reflect univariate changes in brain activity. Fig E: Task-related modulations in regional BOLD activity revealed by GLM analysis. Fig F: Relationship between transfer and learning-related changes in eccentricity. Fig G: Relationship between learning performance and learning-related changes in eccentricity. (DOCX) [file pbio.3003268.s001.docx]

SUPPLEMENTAL INFORMATION

**Fig A:** Comparison of effector- and learning-related changes in manifold eccentricity using different numbers of principal components (PCs).

**Fig B:** Functional connectivity properties that underlie manifold eccentricity.

**Fig C:** Brain regions showing a Task Epoch $\times$ Hand interaction effect.

**Fig D:** Effector- and learning/transfer-related changes in connectivity do not reflect univariate changes in brain activity.

**Fig E:** Task-related modulations in regional BOLD activity revealed by GLM analysis.

**Fig F:** Relationship between transfer and learning-related changes in eccentricity.

**Fig G:** Relationship between learning performance and learning-related changes in eccentricity.


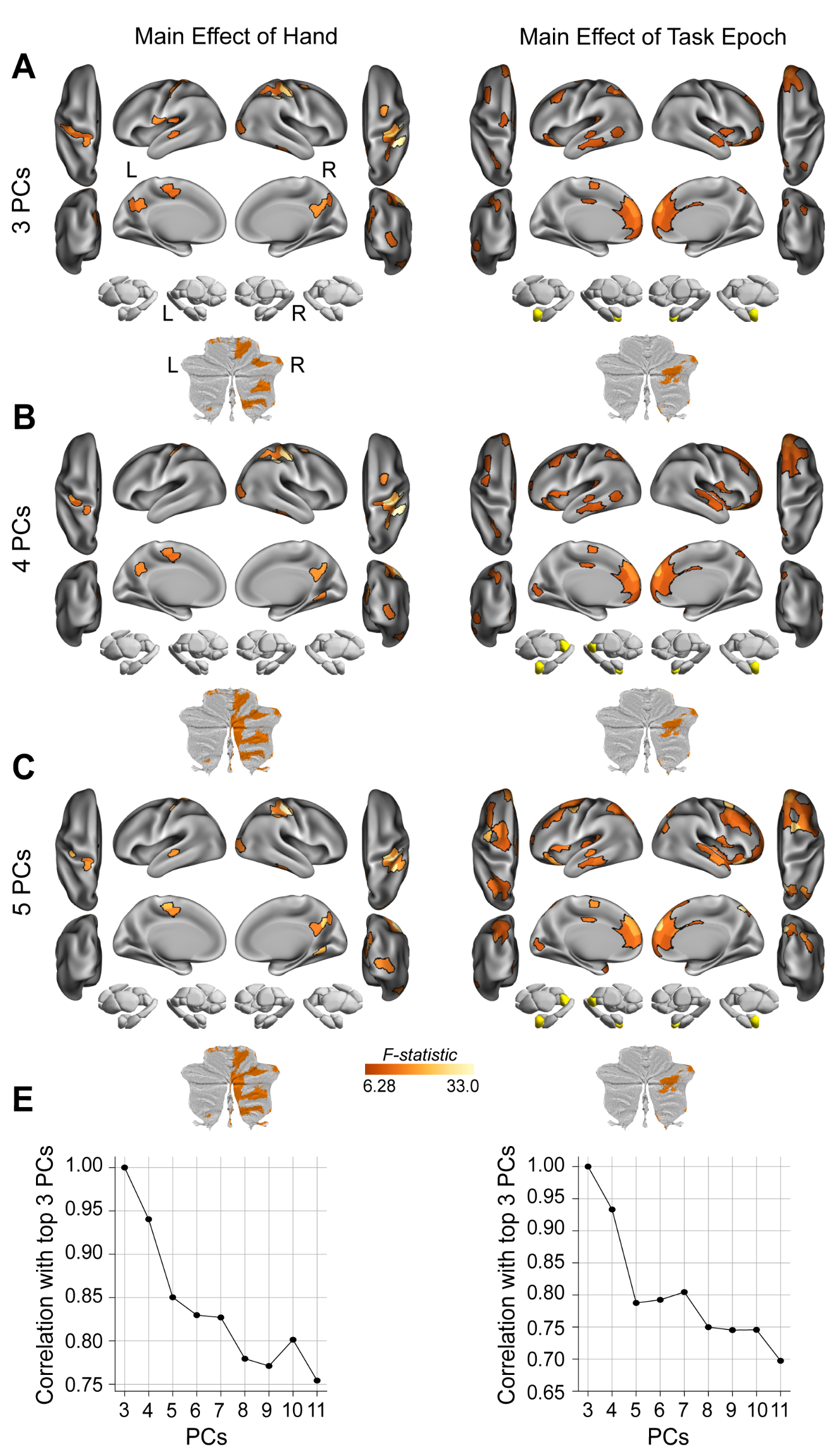


**Fig A: Comparison of effector- and learning-related changes in manifold eccentricity using different numbers of principal components (PCs).** (A-C) The Hand (left) and Task Epoch (right) main effects derived using the top 3 PCs (A), top 4 PCs (B) and top 5 PCs (C). The results show a high degree of correspondence in the significant effects across the different numbers of PCs. (E) Effect of including lower variance-explained PCs on the main effect brain maps. Plots show the spatial correlation between the main effect maps derived from the top 3 PCs (used in the main paper) with the main effect maps that are obtained as additional PCs are included in the analysis (from 4-11 PCs). Note the high degree of spatial correlation, even as additional PCs are added to the data.


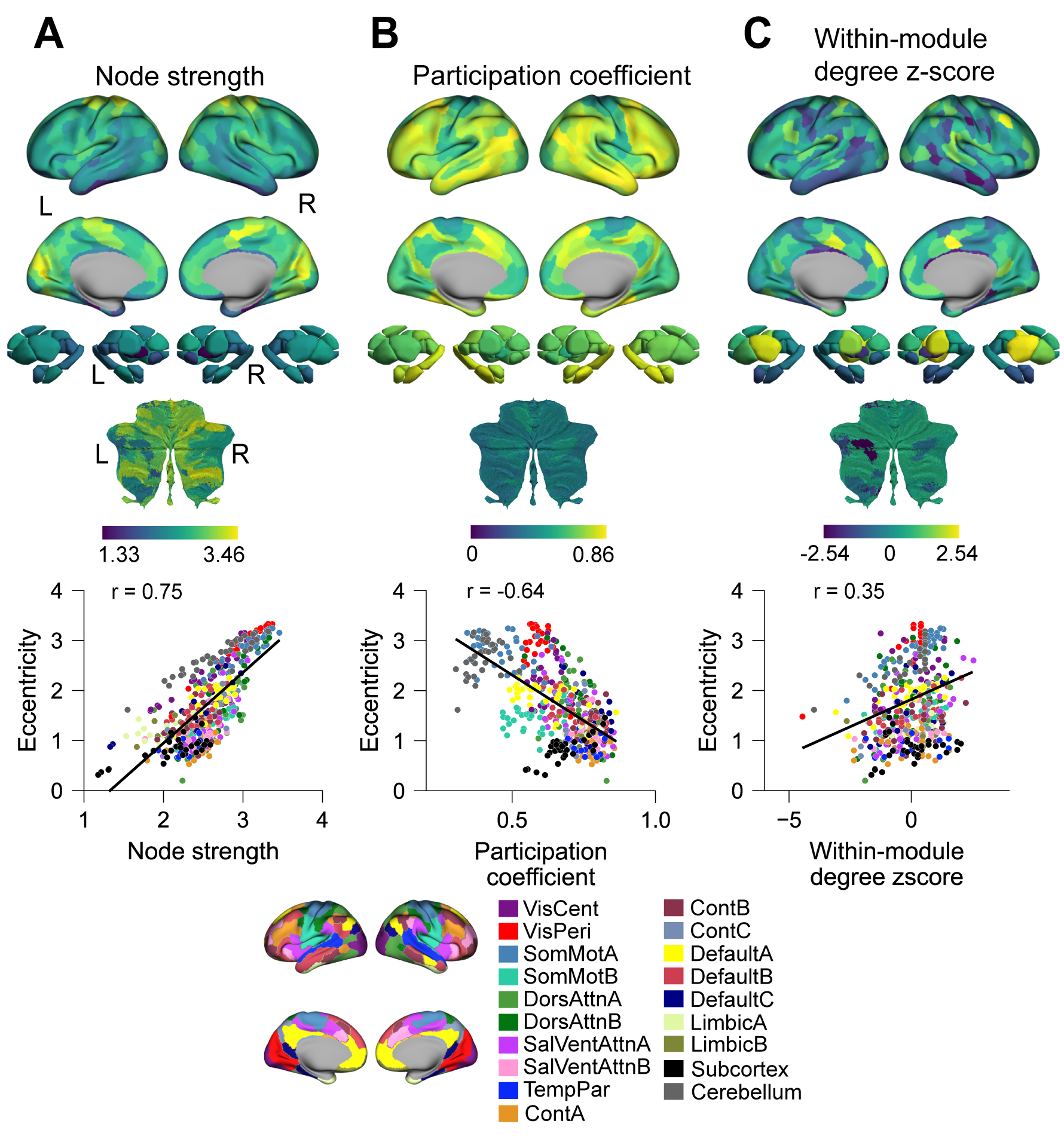


**Fig B: Functional connectivity properties that underlie manifold eccentricity**. (A-C) Different network properties of individual brain areas (derived from functional connectivity) and their correspondence to regional eccentricity. Top, brain plots show the maps of node strength, participation coefficient and within-module degree z-score, derived from the group-average Baseline connectivity matrix (i.e., reference connectivity matrix). Bottom, scatterplots show the relationship between each graph theoretic measure and manifold eccentricity, with the line depicting a regression line of best fit to the cortical, subcortical and cerebellar data. The Baseline eccentricity is positively related to node strength (r = 0.73, two-tailed p < 0.001) and within-manifold degree z-score (r = 0.27, two-tailed p < 0.001), and inversely proportional to a region’s degree of cross-network integration, as measured through participation coefficient (r = −0.33, two-tailed p < 0.001).Together, these results support the idea that areas with higher eccentricity generally have stronger functional coupling with other parts of the same functional network (i.e., higher segregation) whereas areas with lower eccentricity generally have stronger connectivity across different networks (i.e., higher integration).

#
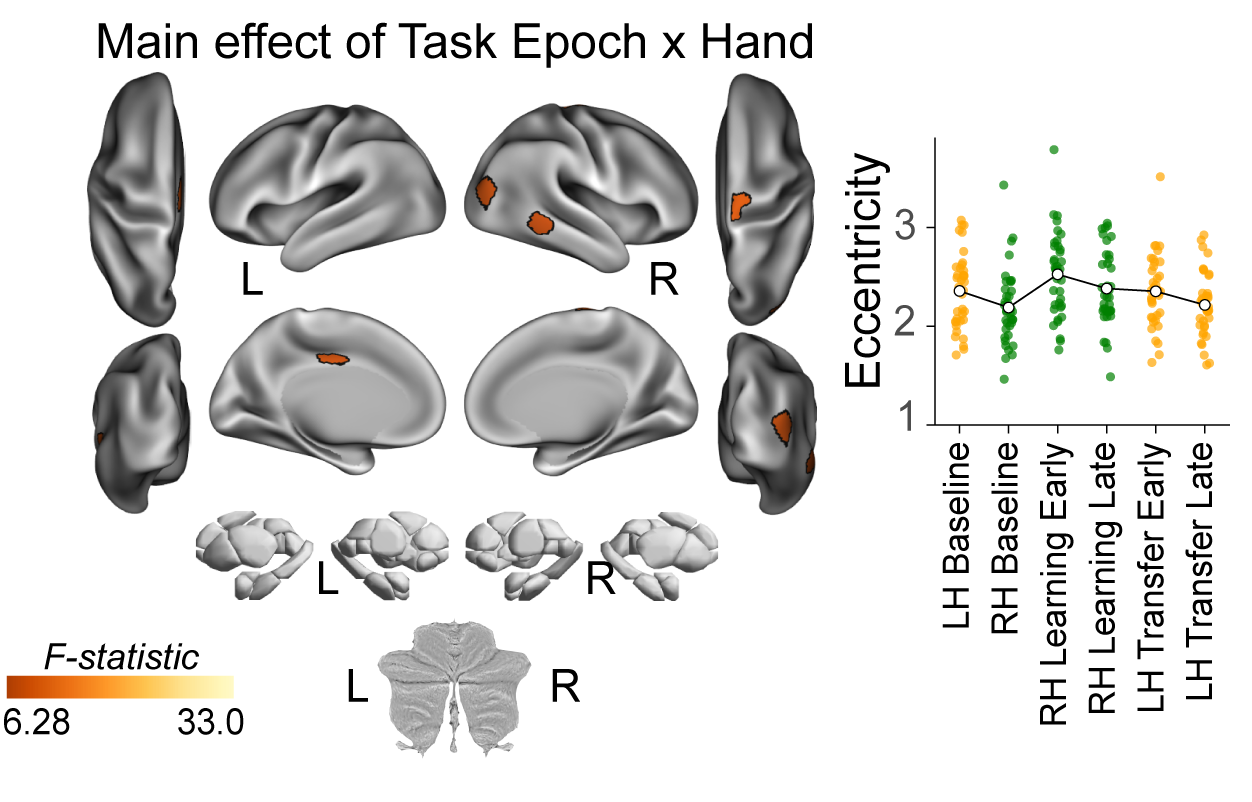


**Fig C: Brain regions showing a Task Epoch** $\times$ **Hand interaction effect.** Left, The four brain regions that exhibited a significant interaction effect based on the region-wise 3 (task epoch) x 2 (effector) repeated measures ANOVAs using a false-discovery rate (FDR) correction for multiple comparisons (q < 0.05). Right, scatter plot shows the eccentricity for each significant region (averaged across participants), with the line plot overlays showing the group mean across task epochs. Note that data points are color-coded, as in Fig 1, according to the hand used during each epoch (orange = left hand; green = right hand). The data and code needed to generate this figure can be found in <https://zenodo.org/records/15648991>.

#
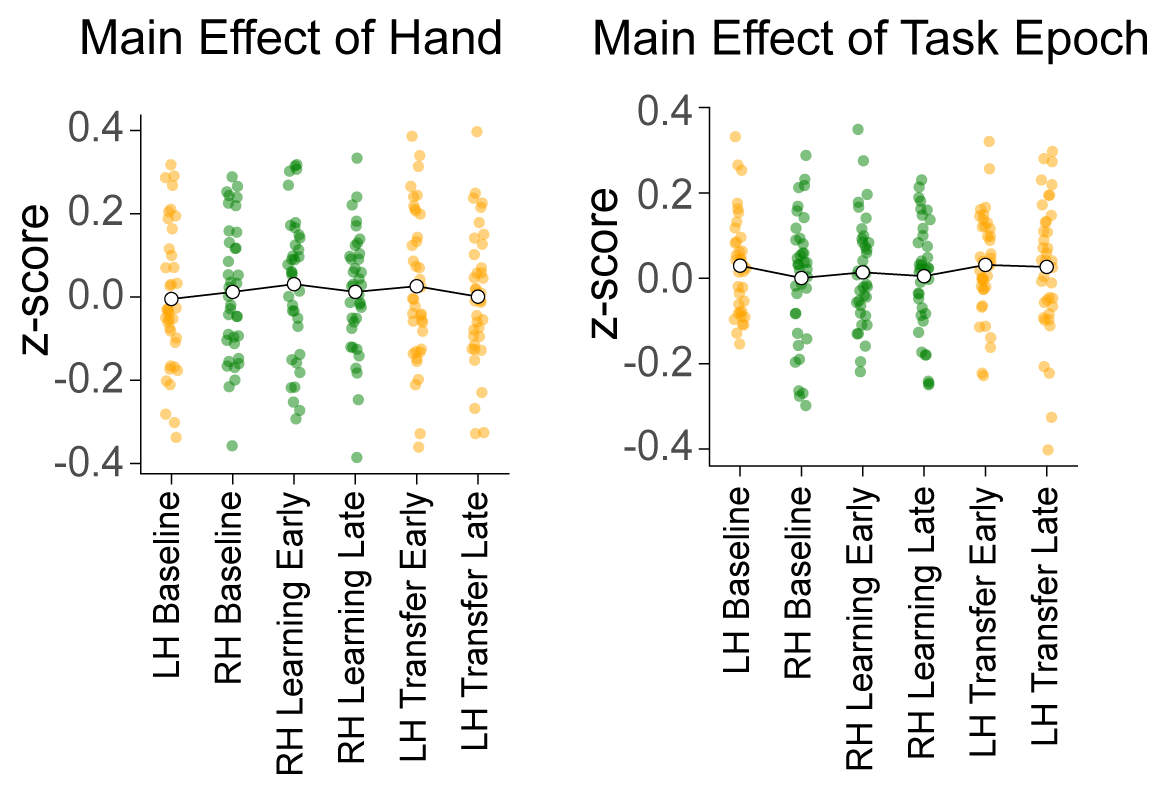


**Fig D: Effector- and learning/transfer-related changes in connectivity do not reflect univariate changes in brain activity.** Plots show the average z-scores of the time series data across epochs for brain regions (averaged across participants) that exhibited a significant main effect of Hand (left) and Task Epoch (right) in the main manuscript. A two-way repeated measures ANOVA with Time and Task Epoch as factors on this z-score data revealed no significant regions after FDR correction (q < 0.05). Thus, the eccentricity changes described in the main manuscript do not appear to reflect underlying changes in BOLD response across epochs. The line plot overlays show the group mean across regions of interest (ROIs) over the task epochs. Data points are color-coded according to the hand used during each epoch (orange = left hand; green = right hand). The data and code needed to generate this figure can be found in <https://zenodo.org/records/15648991>.


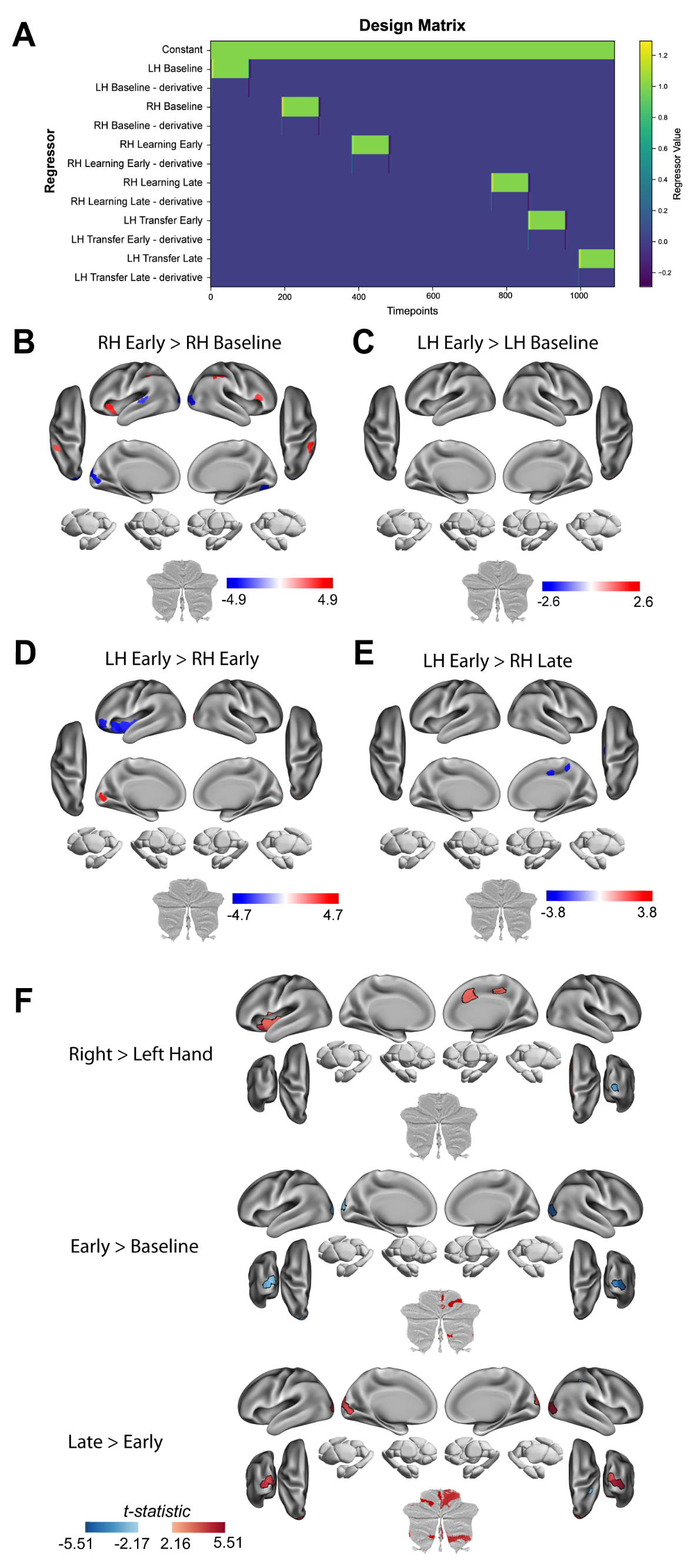


**Fig E**: **Task-related modulations in regional BOLD activity revealed by GLM analysis.** (A) Design matrix used for GLM modeling, convolved with the canonical Glover hemodynamic response function and its temporal derivative. Each regressor corresponds to one of six task epochs spanning baseline, learning, and transfer conditions. (B) Statistical map for the contrast RH Learning Early > RH Baseline, revealing regions selectively engaged during early stages of right-hand learning. (C) Statistical map for the contrast LH Transfer Early > LH Baseline, capturing regions recruited during early transfer to the untrained hand. (D) Contrast LH Transfer Early > RH Learning Early, identifying regions more strongly engaged during transfer compared to initial learning. (E) Contrast LH Transfer Early > RH Learning Late, highlighting regions exhibiting greater activation during transfer relative to late learning stages. All maps display z-scores derived from group-level one-sample t-tests on subject-level beta estimates, thresholded at q < 0.05 using FDR correction. (F) To directly compare with the main functional connectivity analyses (Figs 4 & 5), we performed an identical 3x2 repeated-measures ANOVA (rmANOVA) using the GLM beta estimates, with factors Task Epoch (Baseline, Early, Late) and Hand (Left, Right). This panel shows post-hoc contrasts for regions exhibiting significant main effects in this activation-based rmANOVA. Specifically, Right > Left contrasts are displayed for regions with a main effect of Hand, while Early > Baseline and Late > Early contrasts are shown for regions with a main effect of Task Epoch. This highlights condition-specific BOLD activity modulations analogous to the eccentricity changes examined in the main text.


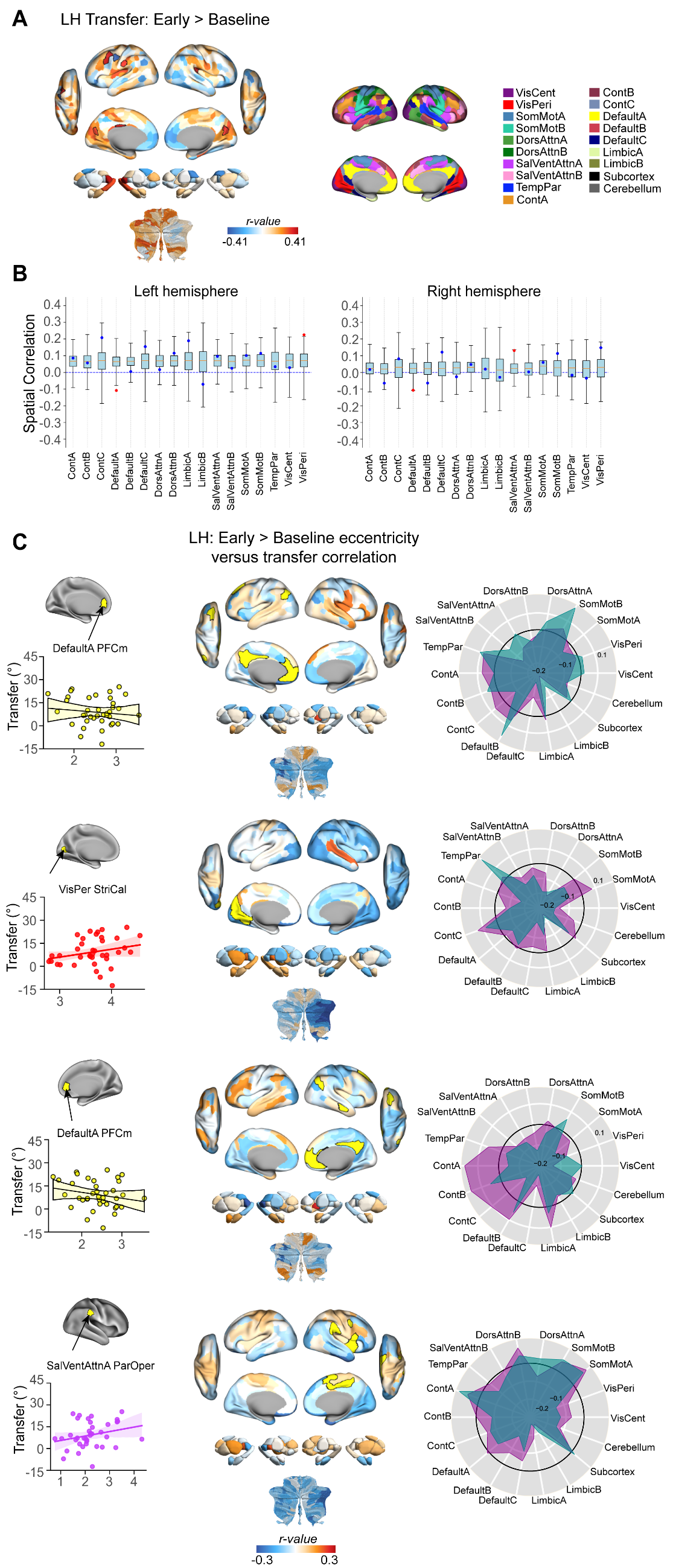


**Fig F: Relationship between transfer and learning-related changes in eccentricity.** (A) Whole-brain correlation map between subjects’ Transfer Rate (from Fig 9B) and the change in regional eccentricity from LH Baseline to LH Early transfer. Black bordering denotes regions that are significant at p<0.05. (B) Results of the spin-test permutation procedure, assessing whether the topography of correlations in A is specific to individual functional brain networks for each hemisphere. Single points indicate the real correlation value for each of the 17 Yeo et al. networks (4), whereas the boxplots represent the parameters of a null distribution of correlations derived from 1000 iterations of a spatial autocorrelation-preserving null model (2, 3). Boxplots are plotted the same as in Fig E. All correlations were corrected for multiple comparisons (q<0.05). The dashed horizontal blue line indicates a correlation value of zero. (C) Left scatterplots show the correlation between the change in eccentricity for a representative region from different significant networks for each hemisphere (denoted in yellow) from LH Baseline to LH Transfer Early with subjects’ Transfer Rate. Middle, underlying pattern of functional network connectivity, and its relationship to learning performance, for each of the significant networks. Positive (red) and negative (blue) values show where an increase in seed inter-network connectivity was associated with either a higher or lower Transfer Rate, respectively (i.e., blue values denote where increased connectivity led to lower transfer errors, or better generalization). Right, Spider plots summarize the patterns of correlation changes at the network-level. Note that the black circle in the spider plot denotes r=0 (i.e., zero correlation between the change in functional connectivity and performance). The data and code needed to generate this figure can be found in <https://zenodo.org/records/15648991>.


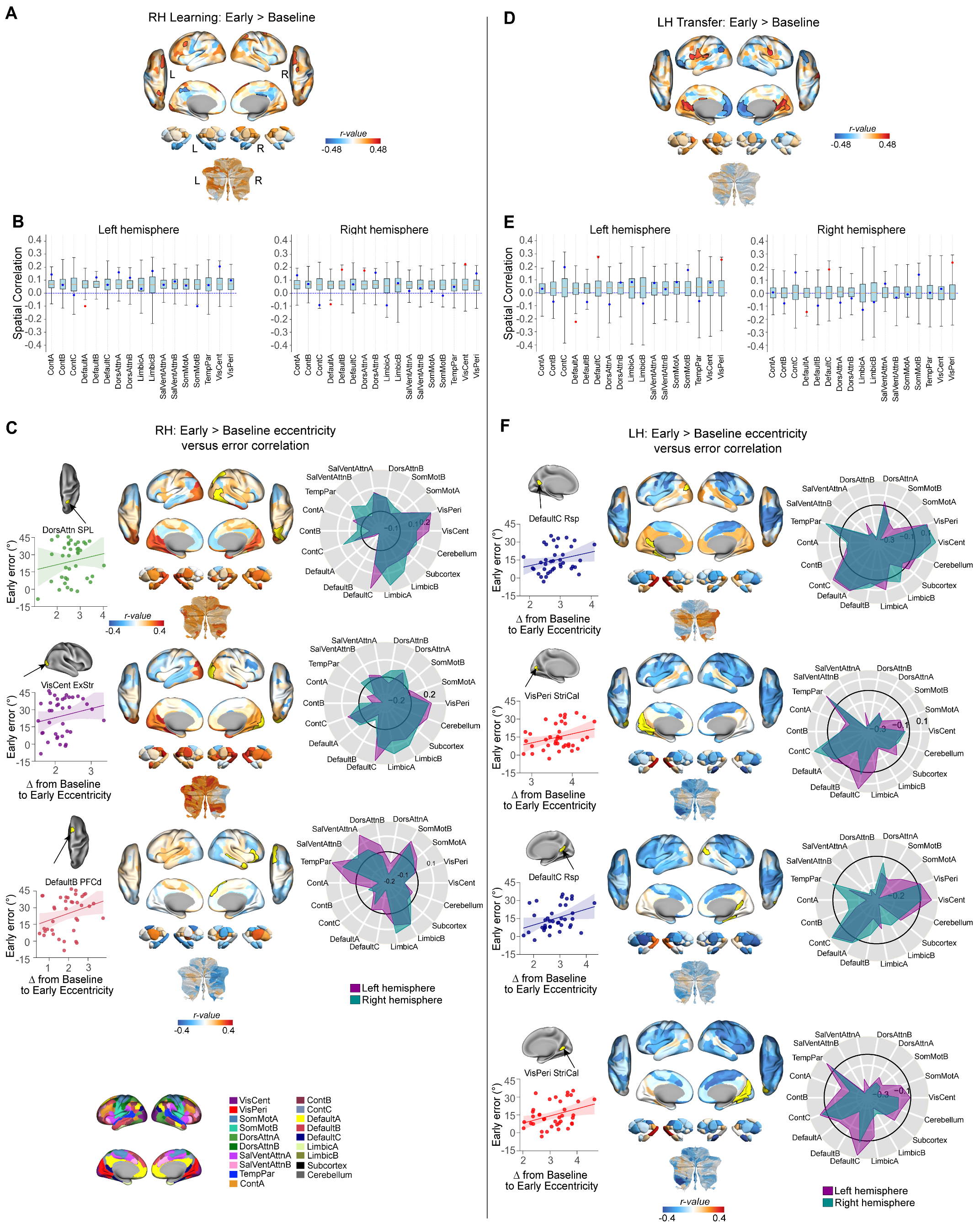


**Fig G: Relationship between learning performance and learning-related changes in eccentricity.** (A) Whole-brain correlation map between subjects’ RH early error and the change in regional eccentricity from RH Baseline to RH Early learning. Black bordering denotes regions that are significant at p<0.05. (B) Results of the spin-test permutation procedure for RH Early Learning, assessing whether the topography of correlations in A and B are specific to individual functional brain networks for each hemisphere. Single points indicate the real correlation value for each of the 17 Yeo et al. networks [(4)](https://paperpile.com/c/UsXXaT/4sC4t), whereas the boxplots represent the parameters of a null distribution of correlations derived from 1000 iterations of a spatial autocorrelation-preserving null model [(2, 3)](https://paperpile.com/c/UsXXaT/sp4NL+PaJBZ). In the boxplots, the ends of the boxes represent the first (25%) and third (75%) quartiles, the center line represents the median, and the whiskers represent the min-max range of the null distribution. All correlations were corrected for multiple comparisons (q<0.05). The dashed horizontal blue line indicates a correlation value of zero. (C) Left scatterplots show the correlation between the change in eccentricity for a representative region from different significant networks for each hemisphere (denoted in yellow) from RH Baseline to RH Early Transfer with subjects’ median angular error during the RH Transfer Early epoch. Middle, underlying pattern of functional network connectivity, and its relationship to learning performance, for each of the significant networks. Positive (red) and negative (blue) values show where an increase in seed inter-network connectivity was associated with either higher or lower angular errors, respectively (i.e., blue values denote where increased connectivity led to lower errors, or better performance). Right, Spider plots summarize the patterns of correlation changes at the network-level. Note that the black circle in the spider plot denotes r=0 (i.e., zero correlation between the change in functional connectivity and performance). (D) Same as A but for the correlation between subjects’ LH early error and the change in regional eccentricity from LH Baseline to LH Early transfer. (E) Same as B, but for the correlation map in D. (F) Same as C, but for the significant networks denoted by the spin-tests in E. The data and code needed to generate this figure can be found in <https://zenodo.org/records/15648991>.
