# Supplementary material for: Transfer of motor learning is associated with patterns of activity in the default mode network
Source: PLoS Biol. 2025 Aug 14;23(8):e3003268. doi: 10.1371/journal.pbio.3003268 (PMC12352662; doi:10.1371/journal.pbio.3003268)
Supplement: Attachment — Submitted filename: Reply to Reviewers.pdf [file pbio.3003268.s004.pdf]

RESPONSE TO REVIEWER COMMENTS

We thank the reviewers for their helpful feedback and suggestions on our manuscript, which have strengthened the interpretability and clarity of our findings. Below, we address each of the comments made from the three reviewers. Please refer to the following legend when reviewing our responses:

(1) Comments from the reviewer are shown in bolded text
(2) Blue text refers to our response to the reviewer's comments
(3) Red text refers to changes in our original manuscript

Reviewer #1: The authors investigated how inter-manual transfers of motor learning, utilizing fMRI and a visuomotor adaptation task. Key findings reveal that the brain's default mode network (DMN), a core of trans-modal areas, reactivates similar activity patterns during the transfer phase as observed during initial learning. These changes are linked to structural properties of the DMN, including myelin content and neurotransmitter receptor density. This research highlights the DMN's critical role in generalizing motor learning across untrained contexts, emphasizing the interplay of higher-order brain areas in sensorimotor learning and generalization.

This paper is distinguished by its well-thought-out experimental design and the use of advanced and diverse techniques for brain data analyses. It effectively captures whole-brain activity changes during motor learning. Their complex analyses are described concisely and clearly, making them easy to understand.

Response:
We thank the reviewer for their positive comments on our paper.

One concern is whether the reactivation of the DMN is specific to intermanual transfer or whether it is a phenomenon commonly observed in the early stages of learning. To answer this question, the relationship between intermanual transfer and DMN reactivation would need to be investigated in more detail.

Response:
We thank the reviewer for raising this important point regarding the specificity of DMN reactivation to intermanual transfer versus whether it reflects a general early learning phenomenon. To directly address this, we have performed a more detailed Representational Similarity Analysis (RSA) to directly compare the eccentricity patterns across different learning and transfer epochs. We have included these new results, and a new corresponding figure (Fig. 6), in the revised manuscript, and also provide these below for the reviewer.

In short, our RSA analyses provide compelling evidence that the patterns of connectivity in DMN areas established during initial RH learning are re-expressed during the LH early transfer phase. In addition, we show that this re-expression effect is specific to DMN areas, as we did not find similar results when we performed the same RSA on brain regions exhibiting a main effect of Hand, primarily localized to sensorimotor cortex.

In our Results section, we have included a new subsection that deals with these results:

"Evidence that the manifold patterns during early learning are re-expressed during transfer

Accompanying the brain map results in Figs. 4E and 5E are scatterplots (rightmost panels) that denote the eccentricity changes of each significant brain region across epochs for each pairwise contrast. In the case of the brain regions showing a main effect of Hand (shown in Fig. 4A), these eccentricity plots clearly delineate the strong contralateral effects described above (and shown in Fig. 4E); i.e., the eccentricity of left hemisphere regions is higher, on average, for RH trials, and vice versa for the right hemisphere regions on LH trials. However, in the case of the brain regions showing a main effect of Task Epoch (shown in Fig. 5A), there are two

important observations to be made. Firstly, unlike the contralateral hand effects (in Fig. 4E), the eccentricity changes in both hemispheres are highly similar (compare left and right hemisphere eccentricity plots in Fig 5E). This indicates that the connectivity changes occurring across the baseline, early and late learning/transfer epochs are largely bilateral in nature, and mainly localized to higher-order, non-motor regions of cortex. Secondly, there is a particularly large increase in the manifold eccentricity of these regions during the first early learning period, which is followed by a second (more moderate) increase in eccentricity during the early transfer period (see Fig. 5E). This second increase in eccentricity, along with the resulting main effect, suggests that the manifold structure observed during initial RH learning may be selectively 're-expressed' during initial LH transfer.

To explore this idea directly, moving beyond simple magnitude comparisons, we used an RSA approach to test whether the spatial pattern of regional eccentricity was similar between the two epochs. Specifically, we tested if the spatial correlation between the pattern of regional eccentricity during the RH early learning and the LH early transfer epochs is significantly higher than the correlation between the RH early learning epoch and the other learning epochs that bookend the LH early transfer epoch (i.e., RH late learning and LH late transfer epochs). This RSA analysis employs a logic similar to that used to measure cortical reinstatement in episodic memory research, where neural activity patterns during encoding and retrieval are directly compared [79–88]. When we performed this analysis, we found that the correlation between the RH early learning epoch and each of the three subsequent epochs significantly differed (repeated measures ANOVA, $F(2,74) = 3.14$, $p=0.049$). Follow-up paired samples t-tests revealed that the spatial correlation between the RH early learning epoch and the LH early transfer epoch was indeed significantly higher than compared to the RH early learning epochs' correlation with each of RH late learning (one-tailed t-test, $t(37) =1.87$, $p = 0.035$) and LH late learning (one-tailed t-test, $t(37) = 2.58$, $p = 0.007$; Fig. 6B) epochs. Together, these findings provide compelling evidence that the manifold structure established during RH early learning is most strongly re-expressed during the early phase of transfer to the untrained left hand.

Note that one alternative explanation of these above results could be that these 're-expression' effects are not specific to higher-order cortical areas *per se*, but rather reflect a brain-wide phenomenon. To explore this possibility, we performed the exact same RSA on brain regions exhibiting a main effect of Hand (in Fig. 4A). We reasoned that these brain areas, primarily localized to sensorimotor cortex and which predominantly showed contralateral effects (Fig. 4E), should demonstrate hand-specific patterns distinct from the re-expression effect observed in the higher-order regions, such as the DMN. Specifically, we would predict that the correlation between the pattern of regional eccentricity during the RH early learning and RH late learning epochs should be significantly higher than the correlation between the former condition and the two LH transfer epochs (early and late). When we performed this RSA on the brain regions exhibiting a main effect of Hand, we again found that the correlation between the RH early learning epoch and each of the three subsequent epochs significantly differed (repeated measures ANOVA, $F(2,74) = 5.08$, $p = 0.008$). Consistent with our predictions, follow-up paired-samples t-tests revealed that the highest correlation with the RH early learning epoch was for the RH late learning epoch, and that the correlations were subsequently lower for both the LH early (one-tailed t-test, $t(37) = -1.84$, $p = 0.037$) and late (one-tailed test, $t(37) = -3.10$, $p = 0.002$) transfer epochs (see Fig. 6D). This contrasting hand-specific pattern in

sensorimotor regions serves as a valuable control, as it further highlights the selective nature of the re-expression effects observed in the higher-order cortical areas, such as the DMN (Fig. 6B).

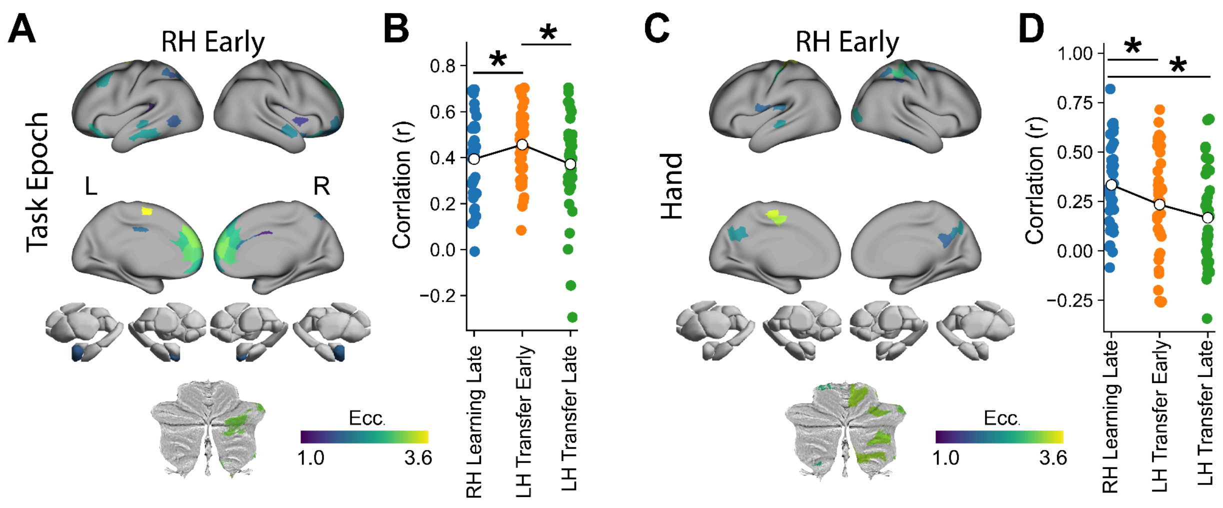

Figure 6: Re-expression of early learning manifold structure across epochs. (A) Mean eccentricity values for the RH early learning epoch for brain regions exhibiting a significant main effect of Task Epoch (i.e., areas from Fig. 5A). (B) Pattern similarity (Pearson r) between RH Learning Early and the three subsequent task epochs—RH Learning Late, LH Transfer Early, and LH Transfer Late—computed across the regions shown in A. The black line shows the across-subject mean, and individual points represent single subjects. (C) Mean eccentricity values for the RH early learning epoch for brain regions exhibiting a significant main effect of Hand (i.e., areas from Fig. 4A). (D) Pattern similarity between RH Learning Early and the same three comparison epochs as in A, but computed across the Hand-significant regions shown in C. * denotes p<0.05 for one-tailed paired samples t-tests."

We believe this new analysis directly addresses the reviewer's main point, and provides compelling evidence for the specific role of higher-order cortical areas, specifically regions of the DMN, in the intermanual transfer of learning.

The authors investigated a correlation between the initial error in the transfer session and the eccentricity of the DMN (Figure 9). However, the initial error in the transfer session alone cannot serve as a direct indicator of intermanual transfer. Ideally, a pretest-posttest paradigm should be used, measuring the initial error with the left hand under visuomotor rotation and then assessing how much this error decreases after training with the right hand under visuomotor rotation.

As the experimental design does not follow this paradigm, an alternative analysis could be to calculate the intermanual transfer rate by comparing the errors between the hands. For example, the percentage increase in the left hand's initial error during the transfer epoch compared to the right hand's final error during the learning epoch could be examined. In this case, a smaller increase in error would indicate a higher degree of inter-manual transfer.

Response:
We thank the reviewer for this insightful comment regarding the measurement of intermanual transfer. We agree that while the initial error during the transfer phase (LH Early Transfer Error) provides a direct measure of performance during the transfer block itself, it is not the only metric for quantifying the degree of learning transfer from the trained (right) hand. Following the reviewer's suggestion, we have now calculated a more direct measure of intermanual transfer for each participant. In our manuscript we have defined this new measure as 'Transfer Rate', which was calculated as the difference between the median angular error during the initial trials of the left-hand transfer block (LH Early Transfer Error; first 32 trials) and the median angular error during the final trials of the right-hand learning block (RH Late Learning Error; last 32 trials). According to this metric, and as noted by the reviewer, lower values indicate better intermanual transfer, reflecting a smaller increase in error when switching to the untrained hand.

We have included this new behavioral measure in our revised manuscript (see new Figure 9B). Importantly, as shown in the new Figure 9C, and perhaps not surprisingly, we found a very strong positive correlation ($r = 0.73$, $p < 0.001$) between subjects' LH Early Transfer Error and this newly calculated Transfer Rate. This strong relationship indicates that subjects who exhibited lower initial errors during the transfer phase were indeed those subjects who demonstrated better intermanual transfer, as defined by the difference relative to their final learning state with the trained hand.

Furthermore, to directly address the reviewer's concern about relating neural changes to this specific transfer metric, we have performed an additional brain-behavior correlation analysis. For this analysis, we correlated the change in network eccentricity from LH Baseline to LH Early Transfer (the same neural measure used in Figure 10E) with individual subjects' Transfer Rate. The results of this analysis are presented in the new Supplementary Figure 6. Critically, these results were qualitatively very similar to those presented in the main text (Figure 10E/F), which used LH Early Transfer Error (which, of course, is to be expected given the strong positive linear relationship highlighted above). In particular, we again identified a significant relationship between the change in eccentricity of the DMN-A network and transfer performance, measured now by the Transfer Rate.

Together, these additional analyses confirm that our primary findings relating DMN-A manifold changes to performance during the transfer phase are robust and hold even when using a different measure (intermanual transfer rate) suggested by the reviewer. Below, we have cut and pasted new excerpts from our manuscript that highlight all of these changes:

In our Results, we now write the following:

"***Changes in whole-brain manifold structure relate to performance***

Up to this point, we have characterized the mean, group-level changes in manifold structure across the learning and transfer phases of the task. Yet, it is well known that subjects can differ markedly in their motor learning abilities [31,33,102,103]. Indeed, while the group-averaged learning curve in Fig. 1B shows that subjects, on average, were able to reduce their visuomotor errors during the task, such learning curves tend to mask significant intersubject variability

(Fig. 9A and B). To help capture the inter-subject variability in transfer performance, and in line with more traditional measures of intermanual transfer [26,54,78,104], we also calculated a 'transfer rate' measure for each participant, defined as the difference between their initial error in the left hand transfer block (LH Transfer Early Error) and their final error in the right hand learning block (RH Learning Late Error). This transfer rate measure reflects the degree to which learning generalizes across hands, with lower transfer error indicating better generalization (Fig. 9B). Perhaps unsurprisingly, we observed a very strong positive linear relationship between subjects' LH Transfer Early Error and this Transfer Rate measure (r = 0.73, Fig. 9C)."

Also, here is the new Figure 9:

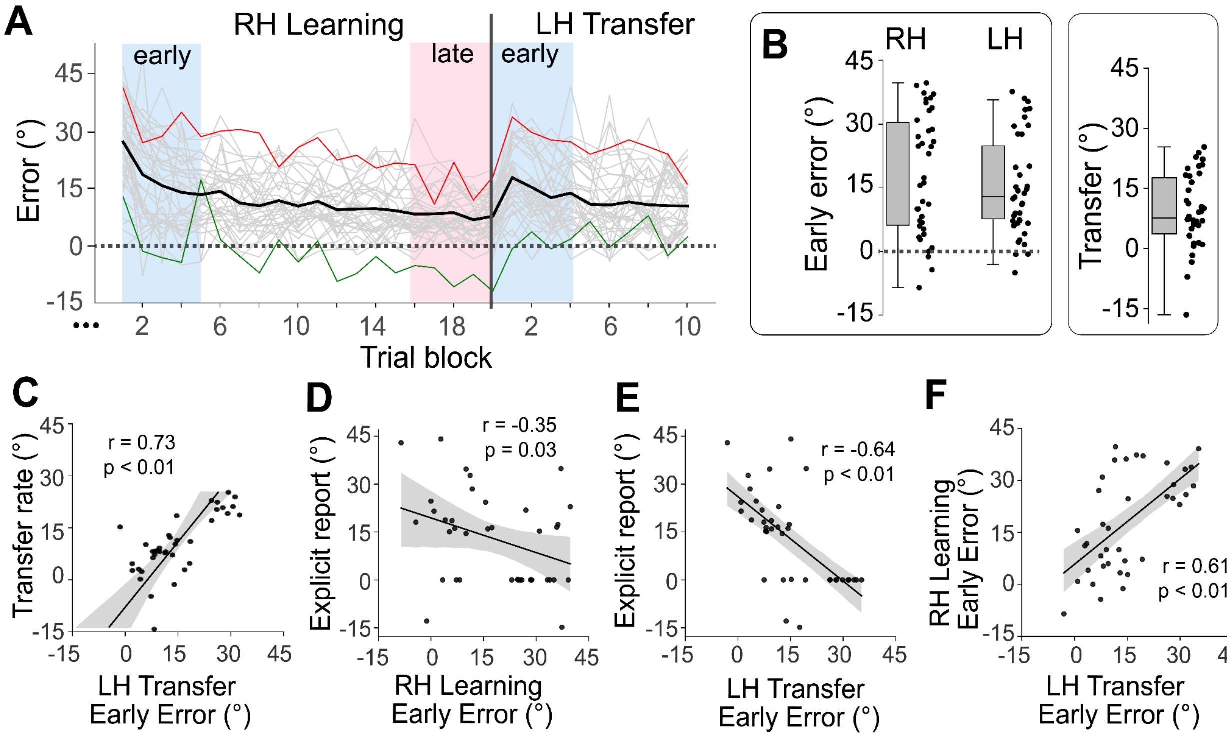

Figure 9: Individual differences in motor learning and generalization. (A) Individual subject learning curves. Solid black line denotes the mean across all subjects, binned by trial block, whereas the light gray traces denote individual participants. The green and red traces denote the learning curves of a good (low error) and poor (high error) learner, respectively. (B) Distribution of median angular error during the RH Early Learning and LH Early Transfer epochs (corresponding to the timepoints covered by the faint blue boxes in A), as well as Transfer Rate, constructed by subtracting the RH Late learning angular error (red box) from the LH Early Transfer epoch error. Single data points denote the median error of individuals. (C) Correlation between subjects' angular error during the LH Early Transfer epoch and Transfer Rate. (D) Correlation between subjects' explicit reports (collected at the end of the Learning block) and angular error during the RH Learning Early epoch. (E) Same as D, but for the

angular error during the LH Transfer Early epoch. (E) Correlation between subjects' angular error during the RH Early Learning and LH Early Transfer epochs. Single data points denote individuals and the black line denotes the best-fit regression line, with shading indicating +1/- standard error of the mean (SEM).

In addition, when introducing our brain-behavior correlation analysis, we now write the following:

"To test this, for each brain region and each of the RH early learning and LH early transfer epochs, we calculated the correlation between subjects' median angular error and the corresponding change in eccentricity from the Baseline to Early epochs for each hand (i.e., RH Learning Early > RH Baseline & LH Transfer Early > LH Baseline, Figs. 10A and D, respectively). [Note that for an analysis of the correlation between the eccentricity change from LH Baseline to LH Transfer Early versus transfer rate, please see Supplementary Fig. 6]."

Separately, when taking the reader through the brain-behavior correlation effects associated with the LH early transfer phase, we also specifically mention the additional supplemental analyses performed with our new measure, Transfer Rate:

"Notably, when we applied the same analyses to the LH early transfer phase of the task, we observed a consistent pattern of effects for the DMN-A network. Specifically, we found that an increase in the eccentricity of bilateral DMN-A networks correlated with better performance during transfer trials (Figure 10E). Additionally, we found significant correlations in other DMN (i.e., DMN-C) and Visual (VisPeri) networks, consistent with the same directionality observed during RH early learning; i.e., less expansion of these networks along the manifold during the early transfer period corresponded to lower error. Further examination of the underlying changes in between-network connectivity associated with the DMN-A network again revealed that enhanced functional connectivity with Control-B and SalVentAttn-B networks was associated with reduced visuomotor errors (Fig. 10F). In addition, we also observed that increased connectivity between the DMN-A and each of the somatomotor and limbic-B networks was associated with lower visuomotor errors. Note that when we performed all the same analyses above but instead using subjects' Transfer Rate as our behavioral measure, the results were qualitatively similar—in particular, re-identifying the unique changes in the DMN-A network and its relationship to performance (see Supplementary Fig. 6). This is to be expected given the strong positive correlation between subjects' Transfer Rate and their LH Transfer Early Error measure (Fig. 9C). Taken together, the consistency of the DMN-A correlation results across both the learning and transfer phases of the task, as well as across both hemispheres, suggest a key role for this particular network in subject-level performance."

Finally, we have also included the new supplementary figure below (Fig. S6), which includes the brain-behavior correlation analyses performed using Transfer Rate:

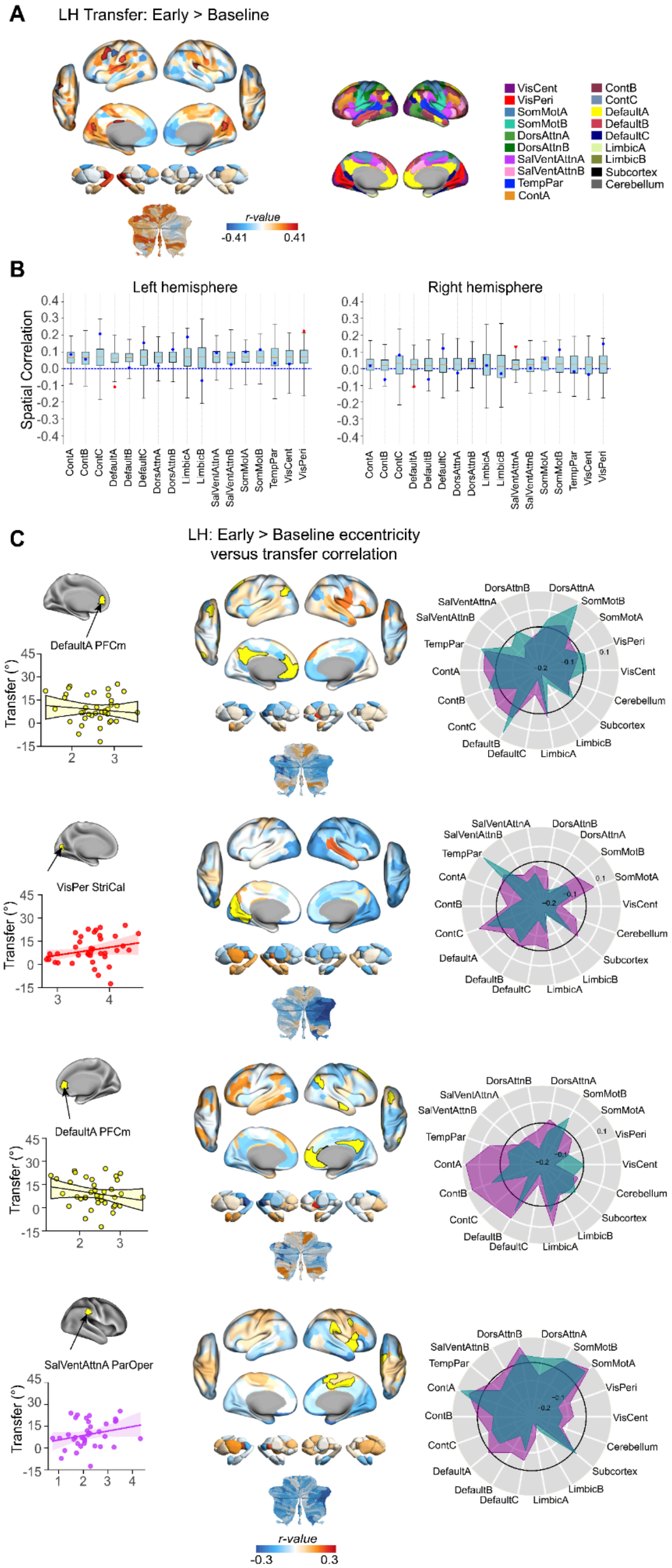
A
LH Transfer: Early > Baseline
r-value
-0.41
0.41
VisCent
VisPeri
SomMotA
SomMotB
DorsAttnA
DorsAttnB
SalVentAttnA
SalVentAttnB
TempPar
ContA
ContB
ContC
DefaultA
DefaultB
DefaultC
LimbicA
LimbicB
Subcortex
Cerebellum
B
Left hemisphere
Right hemisphere
Spatial Correlation
C
LH: Early > Baseline eccentricity
versus transfer correlation
DefaultA PFCm
VisPer StriCal
DefaultA PFCm
SalVentAttnA ParOper
Transfer (°)
r-value
-0.3
0.3

Supplemental Figure 6: Relationship between transfer and learning-related changes in eccentricity. (A) Whole-brain correlation map between subjects' Transfer Rate (from Fig. 9B) and the change in regional eccentricity from LH Baseline to LH Early transfer. Black bordering denotes regions that are significant at $p<0.05$. (B) Results of the spin-test permutation procedure, assessing whether the topography of correlations in A is specific to individual functional brain networks for each hemisphere. Single points indicate the real correlation value for each of the 17 Yeo et al. networks (4), whereas the boxplots represent the parameters of a null distribution of correlations derived from 1000 iterations of a spatial autocorrelation-preserving null model (2, 3). Boxplots are plotted the same as in Fig. S5. All correlations were corrected for multiple comparisons ($q<0.05$). The dashed horizontal blue line indicates a correlation value of zero. (C) Left scatterplots show the correlation between the change in eccentricity for a representative region from different significant networks for each hemisphere (denoted in yellow) from LH Baseline to LH Transfer Early with subjects' Transfer Rate. Middle, underlying pattern of functional network connectivity, and its relationship to learning performance, for each of the significant networks. Positive (red) and negative (blue) values show where an increase in seed inter-network connectivity was associated with either a higher or lower Transfer Rate, respectively (i.e., blue values denote where increased connectivity led to lower transfer errors, or better generalization). Right, Spider plots summarize the patterns of correlation changes at the network-level. Note that the black circle in the spider plot denotes $r=0$ (i.e., zero correlation between the change in functional connectivity and performance).

A minor point is that the cells in the representational similarity matrices in Figures 3C and 3D show values of 0 and 1, which was difficult to understand. If these represent similarity, they should be continuous values. It is unclear whether thresholding has been applied or whether the 0 and 1 values indicate whether statistical significance has been exceeded. No explanation was found in the main text or methods section.

Response:
We thank the reviewer for pointing out this lack of clarity regarding the values shown in the model Representational Similarity Matrices (RSMs) in Figures 3C and 3D. We apologize that our initial presentation was confusing.

The reviewer is correct that RSMs typically display continuous similarity values (e.g., correlation coefficients). However, the matrices shown specifically in Figures 3C and 3D are not empirically derived RSMs from our data; rather, they are theoretical model RSMs that we constructed to test specific hypotheses about the structure present in our actual neural data (shown in Figure 3B).

As indicated by the panel heading "RSM models" (Figure 3C) and "RSM model: Time" (Figure 3D), these matrices represent idealized patterns of similarity based on different conceptual frameworks that may be encoded in the data (i.e., Hand, Epoch, Learning, and Time information). In these model RSMs, the binary values (0 and 1) are used deliberately to represent the predicted similarity structure according to each specific model hypothesis. For instance, a value of 1 indicates that the model predicts the corresponding pair of task epochs/conditions should be similar (i.e., perfectly correlated in the idealized model), whereas a value of 0 indicates that the model predicts the corresponding pair of task epochs/conditions should be dissimilar (i.e., uncorrelated in the idealized model). As such, these binary model RSMs serve as templates against which we compare

our actual, empirically derived, continuous-valued RSM (Figure 3B) using cosine similarity (results of each model is shown in Figure 3E). This allows us to quantify how well each theoretical model captures the representational structure present in the neural data.

To ensure this is clear to all readers, we have revised the legend for Figure 3 and added an additional line of explanatory text in the Results section to explicitly state that Figures 3C and 3D depict theoretical model RSMs (i.e., explaining the meaning of the binary 0 and 1 values as representing predicted dissimilarity and similarity, respectively, according to each model's hypothesis).

For our Figure 3, we now include the following new text:

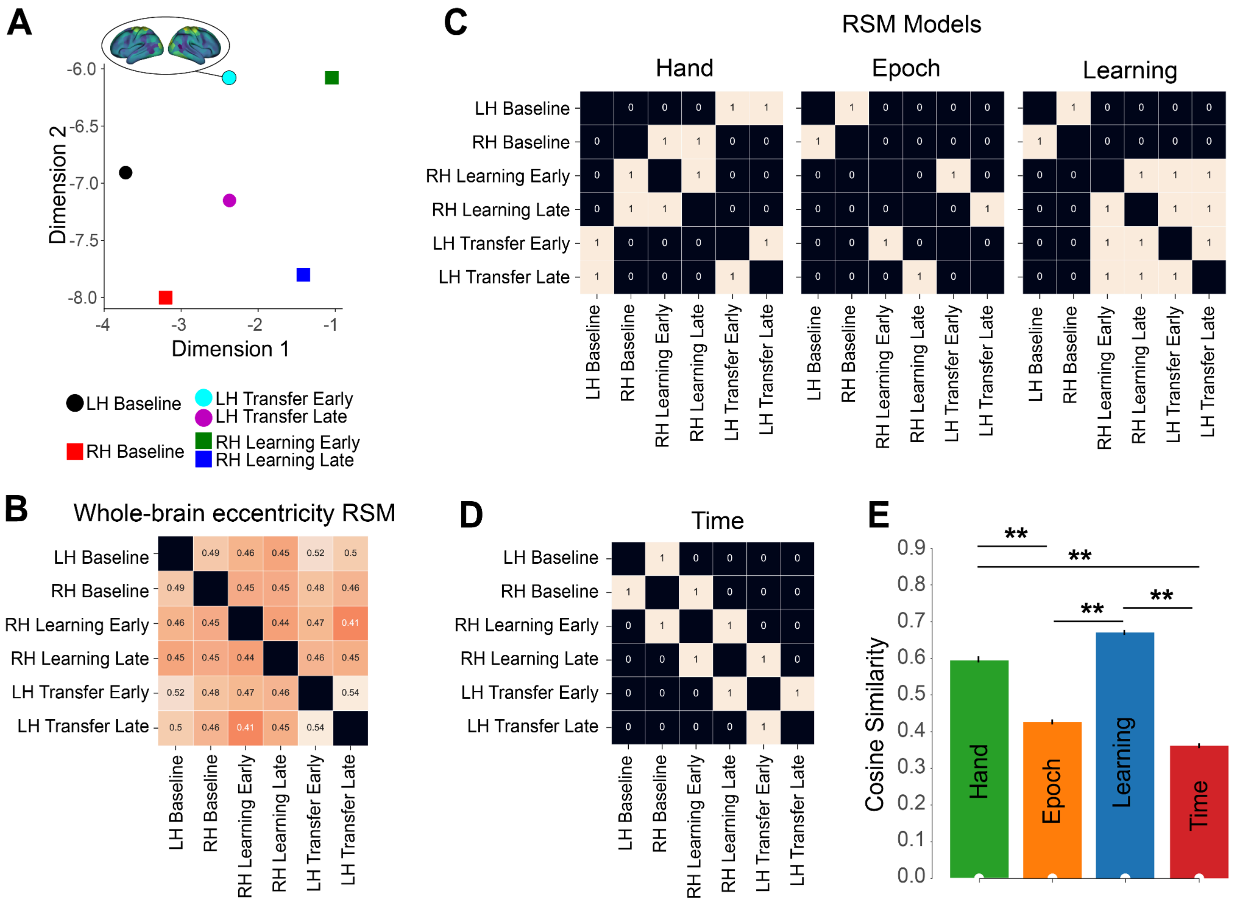

Figure 3: Multiplexing of task-based information in global manifold architecture. (A) UMAP visualization of the similarity in neural states across task epochs (based on the data in B). In this plot, each point represents the across-subject mean whole-brain eccentricity for different task epochs (see legend at bottom), with nearby points representing more similar patterns of whole-brain eccentricity. Note that the two UMAP dimensions appear to capture different types of information about the task structure (see text). (B) Average across-subject representational similarity matrix (RSM) for the patterns of whole-brain eccentricity associated with the six task epochs (the value in each cell represents the Pearson spatial correlation). Note that the RSM is symmetric about the diagonal (black squares, which represent self-correlations). (C) Idealized model RSMs representing different hypotheses about task-related structure. (D) An idealized model RSM representing a control hypothesis based on the passage of time. In these model RSMs (C and D), values of 1 indicate predicted similarity between epochs according to the model, while 0 indicates predicted dissimilarity. These models are compared to the empirical data RSM shown in B. (E) Model comparisons. Each bar indicates the across-subject mean of cosine similarity between the RSM of the whole-brain eccentricity data (in B) and each of the models (in C and D; higher values mean higher similarity). Inference was performed via bootstrap resampling of subjects (1,000 bootstrap samples). Error bars indicate the standard error of the mean. Significance of each model for a one-sided comparison against zero is marked by white dew drops on the horizontal axis, and against the lower-bound estimate of the noise ceiling (gray icicles; FDR-corrected for 4 models). Models' Pairwise differences are summarized by stars. ** denotes q<0.05.

In addition, in the Results main text, we now include the following text:

"To complement these UMAP visualizations and quantitatively test specific hypotheses about the information represented, we performed Representational Similarity Analysis (RSA; [73,74]) on the eccentricity data. Using established methods [75] we constructed, for each subject and epoch, a representational similarity matrix (RSM) by calculating the Pearson correlation between the vectors of regional eccentricity values (464 regions) for every pair of the six task epochs (Fig. 3B shows the group-averaged RSM). This RSM captures the pairwise similarity between the neural states associated with each epoch based on their whole-brain eccentricity patterns.

We next tested how well this empirical RSM structure (Fig. 3B) was explained by three different *a priori* theoretical models representing distinct hypotheses about task structure (Figs. 3C, D): The first model predicted the encoding of hand information only ('Hand' model, Fig. 3C, left), the second predicted the encoding of the task epoch only (Baseline, Early or Late; 'Epoch' model, Fig. 3C, middle), and the third predicted the encoding of the Baseline versus Learning periods only ('Learning model'; Fig. 3C, right). These binary model RSMs represent theoretical similarity structures (where 1 denotes predicted similarity, 0 predicted dissimilarity) corresponding to each hypothesis. We compared each participant's empirical RSM to these theoretical model RSMs using cosine similarity, with higher values indicating a better fit between the model and the eccentricity data. The group-averaged results are shown in Fig. 3E. Statistical inference via bootstrapping revealed that both the 'Hand' and the 'Learning' model performed better than the 'Epoch' model, with the 'Learning' model performing best (though not statistically better than the Hand model; see Fig. 3E)."

Finally, we'd like to thank the reviewer for their thoughtful and encouraging comments on the paper.

Reviewer #2: [NOTE: I reviewed this paper for another journal. My review below echoes some of my previous thoughts, with updates made where appropriate.]

In this study, Rezaei et al. combine functional neuroimaging with a visuomotor adaptation inter-manual transfer task. The authors investigated low dimensional structure in brain-wide connectivity patterns during adaptation, specifically in the case of the transfer of motor learning across hands. The authors were interested in how changes in connectivity structure may be similar (or different) when a learned motor behavior is expressed with an effector that did not undergo initial learning (i.e., effector independence). The authors used cutting-edge fMRI data analysis techniques that allow them to compute patterns of "eccentricity" of particular networks in a low-d connectivity manifold. The authors observed that the structure of these connectivity manifolds was similar across the expression of motor learning for both hands, specifically in areas of association cortex and the default mode network (DMN). This neural connectivity similarity between initial learning and transfer phases was not seen in sensorimotor regions. This result builds upon previous work by this group (Gale et al., '22), linking transmodal and DMN areas to explicit motor learning strategies, which, crucially, are the primary (and perhaps only) driver of inter manual transfer in visuomotor adaptation. Follow-up analyses suggest that the observed neural dissociations may relate to biological/structural properties of cortex, and link DMN connectivity to between-subjects differences in learning and transfer.

Response:
We appreciate the reviewer agreeing to re-review our paper for Plos Biology.

Strengths:
-Overall this is a sophisticated fMRI methodological study and the behavior is nicely characterized. The analysis methods are quite impressive and appear to be rather robust; the neural results are thus generally rather clean and convincing, and multiple control analyses are implemented throughout to help buttress the main empirical claims.
-The addition of a transfer phase in the VMR task is a novel addition that builds upon previous similar work by the group (Gale et al 2022), extending the work to a more direct measure of explicit strategic learning.
-The consistent use of effector-specific (hand-based) analyses throughout the paper helps establish sanity checks and expected "ground truths" (i.e., lateralization effects), on which to support the key learning/transfer related results. This helps with the rigor of the study.
-The addition of subcortical analyses and findings is interesting and broadens the study's scope beyond oft-studied cortical networks/regions.
-The figures and visualizations are generally very thorough, informative, and nicely designed.

Response:
Thanks for these positive comments!

Weaknesses:
- Interpretation/Impact: The two interpretations of the results offered on page 36 - strategy reinstatement and contextual inference - seem like two sides of the same coin (i.e., you have to infer a similar context during early/re-learning to retrieve the correct explicit strategy). Thus it

is not totally clear what the key distinctions are w/r/t interpretations of the data (moreover, the COIN model is focused on implicit motor learning, whereas as here explicit learning is studied). This leads to a more general vagueness, which is that it is not clear how the main DMN connectivity analyses relate to the relevant concepts of strategy formation or memory reinstatement. For the latter at least, there is a large fMRI literature in the episodic memory domain that tackles exactly these questions - i.e., how similar is a multivariate neural pattern in context A (e.g., early learning) versus context B (e.g., late learning). Perhaps by shuffling different learning epochs and looking at multivariate pattern correlations, the authors could do MVPA analyses across DMN regions and actually test for reinstatement-like effects? This would be helpful as it would be a less abstracted analysis of cortical activity and help build more specific interpretations alongside the connectivity results.

Response:
We thank the reviewer for these thoughtful points regarding the interpretation of our findings and the connection to concepts like strategy use and memory reinstatement. We agree that our initial presentation might have framed strategy retrieval/application and contextual inference/reinstatement too much like distinct alternatives, when, as the reviewer correctly notes, they are highly interdependent aspects of generalization.

To address this, we have significantly rewritten the relevant Discussion section to present these processes as integrated facets of a single overarching function supported by the DMN. Specifically, rather than treating them as competing possibilities, we now explicitly frame the DMN's role as integrating context evaluation with strategy reinstatement.

Specifically, in the revised Discussion, we now write:

"This leads to the important question: In what specific ways might the DMN support motor generalization? Based on previous functions ascribed to the DMN [38–40] and its unique neuroanatomical properties described above, one compelling idea is that the DMN performs an integrative function where it is critical for both evaluating the task context and subsequently reinstating the appropriate learned strategy.

First, successful generalization requires recognizing that the current situation—performing the task with the untrained hand—still shares the critical feature (presence of the visuomotor rotation) that necessitates the learned adaptation strategy (i.e., explicit re-aiming). This process of context evaluation is essential; the brain must infer that the previously learned solution remains relevant despite the change in motor effector. Recent computational models, such as the COIN model [35], offer a compelling conceptual framework for how the brain might manage multiple motor memories, indicating that generalization may occur when the brain recognizes a similarity between the current context and the original learning setting. The DMN, with its well-documented roles in memory retrieval, contextual processing, and integrating internal states with external cues [38,41,43,123], seems ideally suited to perform this contextual inference function; i.e., assessing whether the current context warrants deploying the strategy learned during the initial adaptation phase.

Next, once the context is identified as appropriate, the learned strategy must be retrieved from memory and re-expressed. With respect to the implementation of strategies, prior

neurophysiological studies in macaques have implicated DMN neurons in the execution of response strategies during task-based learning [44,124]. This finding accords with the established role of several DMN areas in maintaining high-level, task-based representations for the purposes of planning and problem-solving [125–131]. With respect to memory retrieval, several studies have also implicated several areas of the DMN in this process, suggesting that retrieval involves the reinstatement of neural activity patterns observed during initial encoding [82,84,132–135]. Consistent with this work, we similarly found that the specific pattern of DMN manifold structure observed during early learning was selectively re-expressed during the early transfer phase (Fig. 6). In summary, we posit that the DMN may facilitate both the recognition of the previously encountered context and the retrieval of learned strategies, thereby enabling individuals to generalize their motor behaviours to new contexts or effectors."

We believe that this revised framing directly addresses the reviewer's concern about the interpretations being "two sides of the same coin" by explicitly synthesizing them into a coherent, integrated process. In addition, our new text also clarifies the relevance of the COIN model for the contextual inference aspect. As a small aside — we also want to acknowledge the reviewer's point about the COIN model's initial presentation as often emphasizing tasks involving implicit adaptation. However, we should note that recent theoretical work by the developers of the COIN model (Heald et al., 2023, Annual Reviews Neuroscience) discuss the potential neural implementations of contextual inference, directly speculating about regions like the prefrontal cortex and hippocampus, areas strongly associated with explicit memory and cognitive control. This suggests the core principles of contextual inference might actually bridge implicit and explicit learning processes, potentially providing a mechanism for how the brain determines when to deploy a specific, learned strategy based on contextual cues. Obviously, we don't get into any of this in the paper (as this is probably well beyond its scope), but it is worth mentioning.

Next, regarding the reviewer's suggestion to more directly test for reinstatement using MVPA/RSA on activation patterns — we appreciate this suggestion, as such methods are indeed standard in the episodic memory literature for probing ideas like reinstatement. However, our study's core methodology and primary contribution focus on characterizing macroscale network organization through functional connectivity (covariance) patterns estimated over longer epochs and analyzed via manifold embedding. This approach investigates the architecture of brain-wide communication, which is quite distinct from analyses focused on local activation patterns (e.g., derived from GLM beta coefficients) typically used in activation-based MVPA/RSA. Shifting to a purely regional activation-based MVPA would move the focus away from our central findings concerning system-level connectivity dynamics and manifold structure, and moreover, the current experiment was not designed for such GLM-based analyses (i.e., all trials were spaced 6 seconds apart). Nevertheless, we fully agree on the importance of directly testing for reinstatement-like effects within our data.

To meet the reviewer half-way, we have now performed a new RSA analysis in our paper but which uses the key metric from our connectivity-based framework (i.e., manifold eccentricity). Specifically, we have performed an RSA directly on the pattern of regional manifold eccentricity within the DMN regions identified in our main analysis (i.e., those showing a main effect of Task Epoch). This analysis directly asks: Is the pattern of DMN network embedding observed during

initial learning (RH Early Learning) more similar to the pattern during early transfer (LH Early Transfer) than during other learning/transfer phases?

As detailed in our new Results section ("Evidence that the manifold patterns during early learning are re-expressed during transfer") and shown in the new Figure 6, this analysis provides compelling evidence for reinstatement at the level of network architecture. Specifically, we found that the spatial pattern of DMN eccentricity during RH Early Learning is significantly more correlated with the pattern during LH Transfer Early than compared to the other learning epochs (RH Late Learning or LH Late Transfer). Notably, a control analysis using sensorimotor regions did not show this pattern, highlighting the specificity to higher-order areas like the DMN.

Thus, we believe this new analysis directly addresses the reviewer's core question about reinstatement (a comment also raised by reviewer #1), demonstrating that the network architecture involving DMN regions is indeed re-expressed during transfer, but does so using a method directly consistent with our study's focus on large-scale functional connectivity dynamics.

For the reviewer's convenience, we have included all these new findings, and the associated text, below:

"Evidence that the manifold patterns during early learning are re-expressed during transfer

Accompanying the brain map results in Figs. 4E and 5E are scatterplots (rightmost panels) that denote the eccentricity changes of each significant brain region across epochs for each pairwise contrast. In the case of the brain regions showing a main effect of Hand (shown in Fig. 4A), these eccentricity plots clearly delineate the strong contralateral effects described above (and shown in Fig. 4E); i.e., the eccentricity of left hemisphere regions is higher, on average, for RH trials, and vice versa for the right hemisphere regions on LH trials. However, in the case of the brain regions showing a main effect of Task Epoch (shown in Fig. 5A), there are two important observations to be made. Firstly, unlike the contralateral hand effects (in Fig. 4E), the eccentricity changes in both hemispheres are highly similar (compare left and right hemisphere eccentricity plots in Fig 5E). This indicates that the connectivity changes occurring across the baseline, early and late learning/transfer epochs are largely bilateral in nature, and mainly localized to higher-order, non-motor regions of cortex. Secondly, there is a particularly large increase in the manifold eccentricity of these regions during the first early learning period, which is followed by a second (more moderate) increase in eccentricity during the early transfer period (see Fig. 5E). This second increase in eccentricity, along with the resulting main effect, suggests that the manifold structure observed during initial RH learning may be selectively 're-expressed' during initial LH transfer.

To explore this idea directly, moving beyond simple magnitude comparisons, we used an RSA approach to test whether the spatial pattern of regional eccentricity was similar between the two epochs. Specifically, we tested if the spatial correlation between the pattern of regional eccentricity during the RH early learning and the LH early transfer epochs is significantly higher than the correlation between the RH early learning epoch and the other learning epochs that bookend the LH early transfer epoch (i.e., RH late learning and LH late transfer epochs). This RSA analysis employs a logic similar to that used to measure cortical reinstatement in episodic memory research, where neural activity patterns during encoding and retrieval are

directly compared [79–88]. When we performed this analysis, we found that the correlation between the RH early learning epoch and each of the three subsequent epochs significantly differed (repeated measures ANOVA, $F(2,74) = 3.14$, $p=0.049$). Follow-up paired samples t-tests revealed that the spatial correlation between the RH early learning epoch and the LH early transfer epoch was indeed significantly higher than compared to the RH early learning epochs' correlation with each of RH late learning (one-tailed t-test, $t(37) = 1.87$, $p = 0.035$) and LH late learning (one-tailed t-test, $t(37) = 2.58$, $p = 0.007$; Fig. 6B) epochs. Together, these findings provide compelling evidence that the manifold structure established during RH early learning is most strongly re-expressed during the early phase of transfer to the untrained left hand.

Note that one alternative explanation of these above results could be that these 're-expression' effects are not specific to higher-order cortical areas *per se*, but rather reflect a brain-wide phenomenon. To explore this possibility, we performed the exact same RSA on brain regions exhibiting a main effect of Hand (in Fig. 4A). We reasoned that these brain areas, primarily localized to sensorimotor cortex and which predominantly showed contralateral effects (Fig. 4E), should demonstrate hand-specific patterns distinct from the re-expression effect observed in the higher-order regions, such as the DMN. Specifically, we would predict that the correlation between the pattern of regional eccentricity during the RH early learning and RH late learning epochs should be significantly higher than the correlation between the former condition and the two LH transfer epochs (early and late). When we performed this RSA on the brain regions exhibiting a main effect of Hand, we again found that the correlation between the RH early learning epoch and each of the three subsequent epochs significantly differed (repeated measures ANOVA, $F(2,74) = 5.08$, $p = 0.008$). Consistent with our predictions, follow-up paired-samples t-tests revealed that the highest correlation with the RH early learning epoch was for the RH late learning epoch, and that the correlations were subsequently lower for both the LH early (one-tailed t-test, $t(37) = -1.84$, $p = 0.037$) and late (one-tailed test, $t(37) = -3.10$, $p = 0.002$) transfer epochs (see Fig. 6D). This contrasting hand-specific pattern in sensorimotor regions serves as a valuable control, as it further highlights the selective nature of the re-expression effects observed in the higher-order cortical areas, such as the DMN (Fig. 6B).

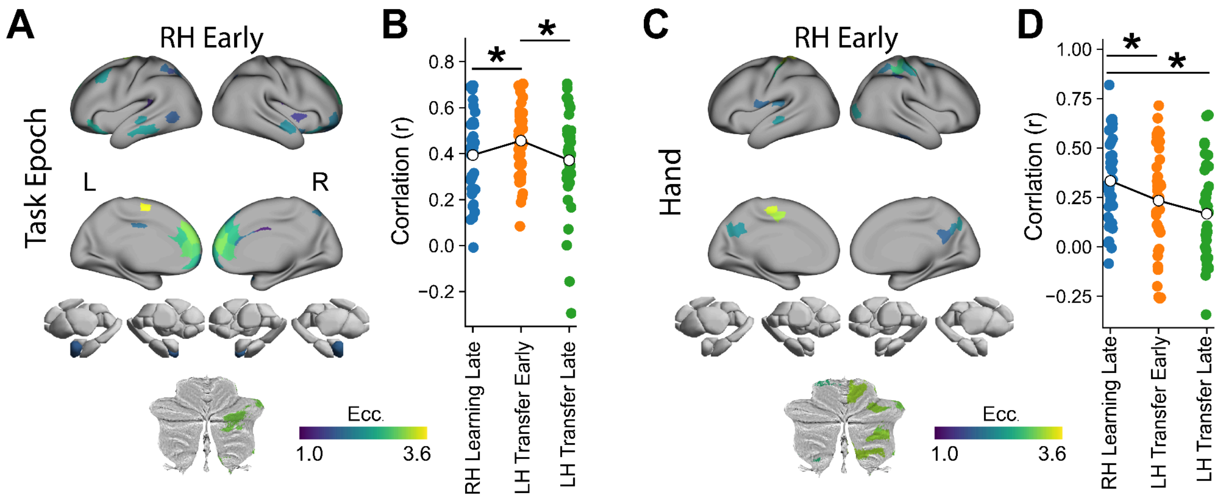

Figure 6: Re-expression of early learning manifold structure across epochs. (A) Mean eccentricity values for the RH early learning epoch for brain regions exhibiting a significant main effect of Task Epoch (i.e., areas from Fig. 5A). (B) Pattern similarity (Pearson r) between RH Learning Early and the three subsequent task epochs—RH Learning Late, LH Transfer Early, and LH Transfer Late—computed across the regions shown in A. The black line shows the across-subject mean, and individual points represent single subjects. (C) Mean eccentricity values for the RH early learning epoch for brain regions exhibiting a significant main effect of Hand (i.e., areas from Fig. 4A). (D) Pattern similarity between RH Learning Early and the same three comparison epochs as in A, but computed across the Hand-significant regions shown in C. * denotes p<0.05 for one-tailed paired samples t-tests."

In summary, we have revised our Discussion to present a more integrated view of the DMN's role, directly addressing the "two sides of the same coin" concern. Furthermore, we have included a new analysis (RSA on eccentricity patterns, Fig. 6) that provides a direct test for the reinstatement of network structure during transfer, consistent with our methodological approach. Collectively, we believe these changes significantly strengthen the interpretation and impact of our findings. Thanks for these suggestions.

-The analyses tend to be quite abstracted from more traditional fMRI measures. These complex, interesting metrics are a feature of the study to be sure, but it would be helpful to have more declarative sentences throughout to really guide the reader about how to think of each result in terms of what are fundamentally higher-order consequences of regional correlations in BOLD time series.

Response:
We appreciate this comment regarding the perceived abstraction of our manifold-based metrics. We recognize the importance of clearly connecting these advanced analyses to the underlying neurobiology and have taken steps to improve this in the revised manuscript.

Specifically, we have introduced a new paragraph early in the Introduction to better motivate and explain the manifold learning approach before presenting our specific hypotheses. This paragraph clarifies why a whole-brain, network-level perspective is valuable for studying distributed processes like motor learning, contrasting it with more localized traditional methods. We explain that manifold learning captures the "holistic organization of brain-wide functional organization" by mapping each region's unique connectivity profile into a common low-dimensional space, representing the brain's "overarching functional geometry." We emphasize that changes in a region's position within this manifold (quantified via eccentricity) reflect meaningful reorganizations of its functional relationships within the global network architecture.

We now include the following text in the Introduction:

"Understanding these processes requires examining not just isolated brain regions, but how large-scale neural systems dynamically coordinate their activity. Traditional fMRI approaches often focus on localized activation changes or connectivity between specific pairs of regions. However, motor learning and generalization engage widely distributed circuits spanning cortical, subcortical, and cerebellar areas [1,7–9]. Investigating changes within such distributed systems

benefits from methods that capture the holistic organization of brain-wide functional organization. To achieve this, we employed manifold learning, a powerful dimensionality reduction technique [10–12]. This approach, which builds upon key observations from both neuronal [10,13–15] and fMRI [16–20] studies, embeds the unique functional connectivity profile of each brain region (its pattern of covariance with all other regions) into a common low-dimensional space. The resulting 'manifold' provides a representation of the brain's overarching functional geometry, revealing the principal axes along which functional network organization varies [12,20]. By analyzing how regions are positioned within this manifold across different task phases, we can track large-scale network reconfigurations – identifying shifts in functional integration and segregation – that underpin learning and its transfer [21–25]."

In addition, in subsequent sections of the Introduction, we now directly link our discussion of putative brain networks (i.e., the DMN) to our analytic measure:

"Given that explicit cognitive strategies are crucial for the transfer of visuomotor adaptation, it is plausible that the DMN uniquely contributes to this process, perhaps reflected by specific changes in its manifold embedding."

And...

"To explore this idea, we investigated changes in the manifold structure derived from functional connectivity patterns across cortical, subcortical and cerebellar brain regions during sensorimotor adaptation and generalization. In our study, human participants learned to adapt their reaching movements during a visuomotor adaptation task performed with their right hand (learning phase), prior to performing the same task with their untrained left hand (transfer phase). To characterize learning- and transfer-related changes at the whole-brain level, using our manifold embedding approach we examined the extent to which different brain regions shifted their connectivity profiles across different phases of sensorimotor adaptation and generalization."

In summary, by introducing and justifying this framework earlier, we aim to provide the reader with a clearer conceptual grounding for interpreting the subsequent results, including metrics like manifold eccentricity, as reflections of large-scale shifts in network organization driven by underlying changes in regional BOLD covariance patterns. We believe this improved motivation and explanation directly addresses the reviewer's concern about the analysis feeling too abstracted from fundamental fMRI measures.

These abstractions also made me wonder how much within-run changes in gross univariate BOLD signal could affect the manifold/eccentricity metrics via more low-level statistics, like signal increases or even changes in the SNR? For instance, early learning/transfer are cognitively more demanding than other epochs, which would have large univariate effects throughout the brain.

Response:
The reviewer raises a valid and important concern regarding the potential influence of gross changes in univariate BOLD signal on our connectivity-based manifold metrics. Indeed, its important to consider whether observed changes in manifold structure, such as eccentricity shifts,

reflect genuine alterations in the covariance patterns between regions, rather than simply echoing overall increases or decreases in regional activity levels that might occur.

To address this point, we had performed a dedicated control analysis in our original paper (*note that we had included this analysis due to the previous comments we received from this reviewer for another journal). In this analysis, we examined the mean BOLD signal amplitude (using the z-scored time series data that formed the basis of our connectivity calculations) for the brain regions that showed significant main effects in our primary analyses (i.e., main effect of Hand, Fig 4A; main effect of Task Epoch, Fig 5A). We compared these mean activation levels across the different task epochs (Baseline, Early, Late) using the same statistical approach (two-way repeated measures ANOVA) employed for our main eccentricity analyses.

As detailed in Supplementary Figure 4 (shown below) and briefly mentioned in the main Results text, this analysis revealed no significant differences in mean BOLD signal amplitude across the task epochs for the regions exhibiting significant manifold changes.

In the results we had wrote:

"When compared to the much larger number of brain regions exhibiting a main effect of Task Epoch (51 regions in total), this suggests that the majority of brain areas displayed similar patterns of manifold eccentricity across both the RH learning and LH transfer epochs (Baseline, Early and Late). ***Notably, further analyses on the underlying time series data revealed that these changes in manifold eccentricity did not reflect relative increases versus decreases in the BOLD response across different epochs (see Supplementary Fig. 4)***."

And, below we present that corresponding supplementary Figure:

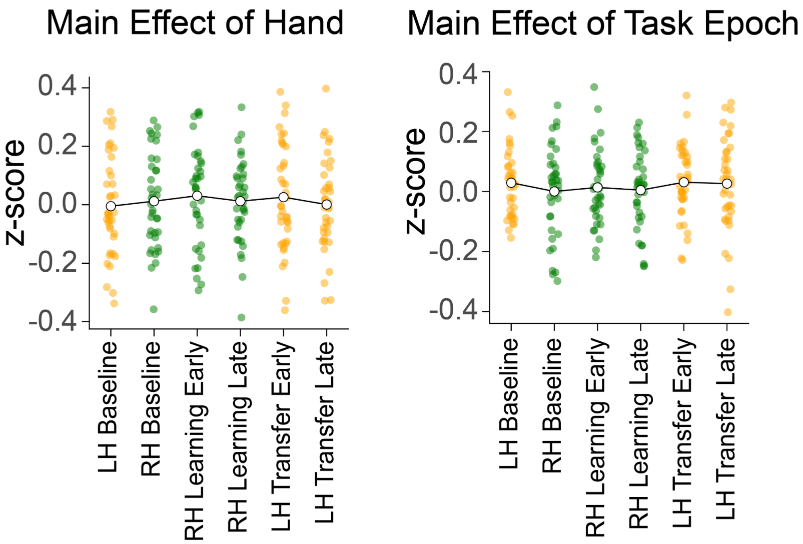

Supplementary Figure 4: Effector- and learning/transfer-related changes in connectivity do not reflect univariate changes in brain activity. Plots show the average z-scores of the time series data across epochs for brain regions (averaged across participants) that exhibited a significant main effect of Hand (left) and Task Epoch (right) in the main manuscript. A two-way repeated measures ANOVA with Time and Task Epoch as factors on this z-score data revealed no significant regions

after FDR correction (q < 0.05). Thus, the eccentricity changes described in the main manuscript do not appear to reflect underlying changes in BOLD response across epochs. The line plot overlays show the group mean across regions of interest (ROIs) over the task epochs. Data points are color-coded according to the hand used during each epoch (orange = left hand; green = right hand).

This control analysis above provides critical evidence that the significant shifts in manifold eccentricity reported in our main findings (Figures 4 & 5) are not merely driven by, or epiphenomenal to, changes in the overall BOLD activation levels within these regions. Instead, they appear to reflect true reconfigurations in the patterns of functional connectivity (covariance) between brain areas during adaptation and transfer. We again appreciate the reviewer raising this point, as it underscores the importance of this control for interpreting our connectivity-based findings.

-It seems possible that other more task-positive networks could have shown up in these analyses, like the "Action Mode Network" (similar to the CON network, Dosenbach et al 2025). It would be interesting for the authors to discuss not just why the DMN showed up, by why more task-oriented networks did not, given the active, externally-focused nature of strategic motor learning.

Response:
The reviewer raises an excellent point regarding the seemingly less prominent role of task-positive networks in our manifold analyses, particularly given the active (and explicit) nature of strategic motor learning. We believe that several factors could contribute to this observation.

Firstly, our analysis specifically focused on manifold structure and *changes* in connectivity patterns observable at a whole-brain level. While task-positive networks are undoubtedly crucial for the execution and online control of motor actions during both learning and transfer, their contribution to the learning and generalization aspects, particularly as captured by manifold reorganization, may manifest differently compared to the DMN and other higher-order regions of cortex. Indeed, it's possible that task-positive networks are consistently engaged throughout the entire task for motor execution and error correction, exhibiting more stable connectivity patterns across the baseline, learning and transfer phases, rather than the reconfiguration and re-expression of manifold structure we observed in the DMN. In this way, their baseline level of engagement and connectivity might be high and necessary for the task, but the *change* in their connectivity pattern specifically related to generalization might be less pronounced (or captured differently by manifold analysis) compared to the DMN. Of course, we should also note that, for the main effect of Task Epoch, while it is true that the DMN subnetworks occupied the #1 and #2 spots in terms of % significant regions, the Control-B subnetwork held the #3 spot (see Fig. 5A, B panels below):

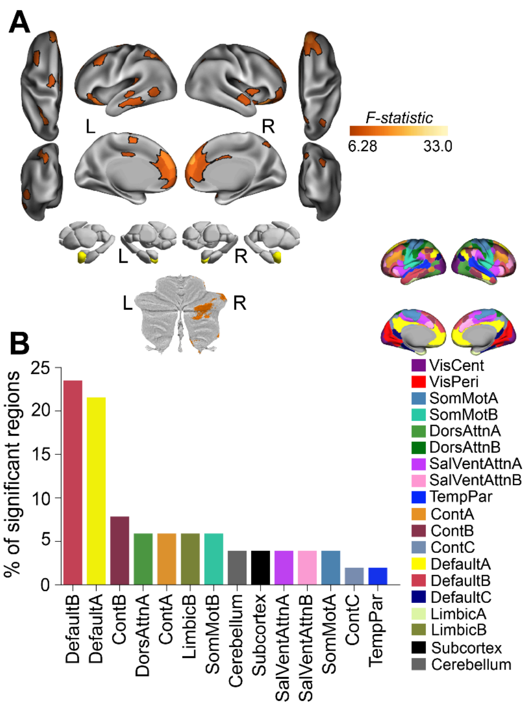

Secondly, the DMN's involvement, as we have argued (and as emphasized in our new Discussion section, cut and pasted above), may be particularly critical for the explicit, strategic component of motor generalization. While task-positive networks are essential for many aspects of task performance, the DMN's unique neuroanatomical and neurophysiological properties, as discussed in our paper (and earlier in our reply), may render it particularly well-suited for supporting the higher-order cognitive processes involved in the contextual inference and strategy implementation crucial for generalization. The 'Action Mode Network' mentioned by the reviewer (from Dosenbach et al., 2025), which is related to goal maintenance and action planning, might indeed be active during the task, but its role in generalization itself, as opposed to task execution, might be distinct from the DMN's.

Thirdly, it's important to consider the nature of our manifold analysis. By focusing on whole-brain connectivity patterns and dimensionality reduction, we may have preferentially captured network-level changes that are more distributed and involve higher-order integration, which are hallmarks of DMN function. Task-positive networks, while crucial, might exhibit more localized or pairwise connectivity changes that are less prominent when viewed from a whole-brain manifold perspective (indeed, Dosenbach et al., 2025 focus on the particularly strong coupling between the AMN and somatomotor network). Notably, we have a current study in our lab focused on directly examining functional coupling between the AMN and somoatmotor network that will hopefully directly address the reviewer’s questions, and thus gain a more complete picture.

Finally, it's worth noting that the observed effects are not mutually exclusive. Task-positive networks and the DMN may work in concert during motor learning and generalization, albeit with potentially distinct roles. Task-positive networks might be crucial for the execution and control aspects, while the DMN contributes to the strategic, cognitive, and contextual facets of generalization. In any case, our findings highlight the previously underappreciated role of the DMN in motor generalization, and further research is needed to fully delineate the specific contributions

and interplay of both task-positive and DMN networks in supporting this complex cognitive-motor ability.

Prompted by this reviewer's query, we have now added a new paragraph to our Discussion section where we directly address this important topic:

"Given the role of strategy use in motor learning and generalization [27–29,31,33,78], another pertinent question is why other task-oriented networks, such as the Action Mode Network [136] or the Control network [71], were not as prominently featured in our analyses as the DMN. One possibility is that this apparent discrepancy reflects the specific nature of our manifold approach, designed to capture relatively large reconfigurations in whole-brain connectivity architecture across learning phases. It is conceivable that brain regions located in task-positive networks, while clearly essential for the moment-to-moment execution of motor actions and online error correction [136–139], might maintain a consistently high baseline of engagement and connectivity across the baseline, learning and transfer phases of the task. Consequently, any changes in their connectivity patterns, as captured by manifold reorganization, might be less prominent compared to those observed in the DMN, where we found a notable re-expression of manifold structure during transfer. It is important to note, however, that task-positive networks were not entirely absent from our analyses; for instance, the Control-B network did rank as the third most modulated for the Task Epoch effect (Fig. 5B), indicating some degree of involvement. Future investigations, perhaps employing methods sensitive to finer-grained temporal dynamics or regional activation patterns, could further elucidate the distinct contributions and interplay of both DMN and task-positive networks in supporting the multifaceted demands of motor learning and generalization."

Minor Things:
-There is a sentence repeated before the last paragraph on page 35

Response:
Thanks for catching that. We have removed that repeated sentence.

-Sometimes figure panels are cited in odd places relative to the flow of the text (e.g., fig 4D). These could be cited earlier or moved to a lower part of the figure?

Response:
We appreciate where this comment is coming from, but wanted to have this panel D appear in the figure in a location close to panel A (on which the meta-analysis is based). We would prefer to keep this arrangement as is if that is okay with the reviewer. However, note that, as a consequence of adding in our RSA-related re-expression analyses (Figure 6), we have moved up the meta-analysis section in our text, such that it is now located closer to when the figure is first described. This will certainly help with the flow of the text.

-It is not exactly clear what the word-cloud analyses add? These could be motivated more.

Response:

In response to your comments in the previous version of our manuscript that you reviewed, we had added several new bits of text to the word-cloud analysis section to make it clear our motivation for the analysis (we recognized that if you had these questions about our paper, then others would as well, and we wanted to revise the text accordingly when we submitted to Plos Biology). Below, we highlight (in bold text) the main textual changes that we included in our submission.

*"To probe the putative behavioral-cognitive processes supported by the distinct patterns of brain activity identified above, we performed a meta-analysis on the regional eccentricity maps in Figs. 4A and 5A using the Neurosynth database [73] and NiMARE library [72]. **This meta-analytic decoding approach allows us to interpret the functional significance of our neural findings by linking the observed patterns of brain activity to cognitive processes identified in the broader neuroimaging literature. By doing so, we can gain insights into the potential behavioral and cognitive functions associated with the regions showing significant changes in eccentricity, thereby enhancing our understanding of the underlying processes supporting motor learning and generalization.***

...further on, we write the following:

*"By contrast, for our main effect of Task Epoch F-stat map (in Fig. 5A), we find that this brain map is positively correlated with terms like 'social' and 'mental states', as well as several other higher-order cognitive processes (e.g., 'theory of mind'); whereas it is negatively correlated with terms like 'movements', 'motor imagery' and 'action' (Fig. 5D). **This suggests that the regions involved in learning and generalization are linked to higher-order cognitive functions rather than traditional motor functions.** At first glance, this outcome may seem counterintuitive given that the Task Epoch F-stat map describes neural changes associated with motor learning and generalization. However, it is generally consistent with contemporary work positing that explicit cognitive processes, which underpin many abstract higher-level functions (e.g., theory of mind), also play a critical role in motor learning and generalization [10,13,74]. **Thus, these analyses not only corroborate our findings by associating motor-related brain maps with motor functions but also reveal that the regions involved in learning and generalization are linked to higher-order cognitive processes.**"*

We trust that, with these additions to the paper, the motivation of these analyses is much clearer.

Finally, thanks for your thoughtful and encouraging suggestions regarding our paper!

Reviewer #3: This is fascinating, and methodologically very elaborate, human neuroimaging study of transfer of motor learning (from right to left hand) using sensorimotor adaptation task. The functional connectivity analysis across different epochs of the task suggests that the transfer of learning across the hands is supported by similar patterns of activity as those that underlie the initial learning, in the "default mode network" (DMN) regions.

There is much to like about the study. The Introduction and Discussion are extremely clear and very much to the point. Generally, the paper is very well written. The methods are highly elaborate and well documented, and as much as I am able to judge various pipelines, very rigorous. The results, taken at face value, are very interesting. However, I found the presentation of results extremely dense and at times too abstract, making it very hard to follow and evaluate the main messages of the study. In addition, I have a number of methodological comments.

Response:
We thank the reviewer for their positive feedback on our paper. Below, we address each of their comments, point by point.

Specifically, I am listing four main topics in terms of clarifications/questions, which are primarily related to the issues of understanding and possibly interpretation. But I think it would be important to clarify these concerns also for the benefit of a broad readership of the PLoS Biol.

1) The nature of the task. Given what is probably highly noticeable dissociation between intended and observed movements (45 deg rotation), this is arguably a case of very explicit adaptation, at least in those subjects who showed explicit re-aiming in the report trials. Why so large rotation has been used?

Response:
We thank the reviewer for raising this point about the task parameters. The reviewer is correct that a 45° visuomotor rotation is substantial and, as intended, strongly promotes the engagement of explicit adaptation strategies, particularly in participants who become aware of the manipulation (e.g., Taylor et al., 2014, de Brouwer et al., 2018; 2022).

Our choice of this rotation magnitude was deliberate and directly motivated by the specific goals of our study. Seminal behavioral work has established that the intermanual transfer of visuomotor adaptation (i.e., generalization from the trained to the untrained hand) is predominantly, if not exclusively, driven by the transfer of explicit cognitive strategies, rather than implicit motor recalibration (Bouchard & Cressman, 2021; Werner & Struder, 2022; Poh et al., 2016). Given our central hypothesis that the DMN, a network heavily implicated in higher-order cognitive functions, plays a key role in supporting the cognitive processes underlying motor generalization, it was crucial to employ a task paradigm that would robustly elicit these explicit strategies known to drive transfer.

While smaller rotations (~10-30°) have been shown to primarily engage implicit processes (e.g., Morehead et al., 2015; Bond et al., 2015), the 45° rotation ensures that, at least in a significant proportion of subjects, that the explicit system is engaged, providing the necessary conditions to

investigate its neural correlates (including DMN activity and connectivity) during both initial learning and subsequent generalization via intermanual transfer. Therefore, this parameter choice was optimal for testing our specific hypotheses regarding the role of higher-order cognitive networks like the DMN in the transfer of learning.

To further clarify this rationale within the manuscript, we have now added a brief justification to the Methods section where the task parameters are introduced.

"...Following a series of baseline trials with each hand, we introduced a 45° clockwise (CW) rotation to the viewed cursor to investigate learning with the right hand. We selected this rotation magnitude specifically because it is known to robustly engage explicit adaptation strategies [27,31,33], which are the primary drivers of intermanual transfer in this paradigm [54,78], thus allowing us to effectively investigate the neural correlates of generalization."

A related concern with the task is that 45 deg rotation apparently matches 45 deg between 8 possible target positions around the circle, potentially eliciting a high level abstract strategy to aim for the "preceding counterclockwise" target. Was there any debriefing of participants, outside of report trials, and have authors considered this possibility?

Response:
The reviewer raises the point about the potential for participants to adopt a specific high-level abstract strategy, such as aiming for the preceding counterclockwise target, given the alignment of the 45-degree rotation with the target positions. Though we did not conduct formal post-experiment debriefing beyond the report trials, this does not seem a plausible approach. It is important to note that the eight target locations were not presented *simultaneously*, and the order of targets was pseudorandomized within each block of eight trials. This sequential and pseudorandom target presentation makes it unlikely that participants could simply aim directly at a visually present 'preceding' target. Instead, if participants were employing a strategy related to target location, it would likely involve a more abstract cognitive transformation of the target vector (i.e., strategic re-aiming), rather than a direct aiming shift to a physically visible alternative target.

We have now added a sentence to our Methods section where we better clarify the sequential and pseudorandom target presentation. We now write the following:

"...The target appeared at one of eight randomized locations, separated by 45° increments (0, 45, 90, 135, 180, 225, 270, and 315°), in bins of eight trials. Note that the targets were presented sequentially, one per trial, and their order pseudorandomized within these 8-trial bins; this prevented participants from using a simultaneously visible adjacent target as a direct aiming guide and ensured that any strategy involving aiming towards a different location required a cognitive transformation of the current target goal."

I really like the analysis in Figure 8. At least in the subjects that showed some rotation awareness and explicit strategic adjustment, it makes sense that self-referential functions supported by DMN are engaged. But it was not readily clear how to relate the inter-subject differences in explicit learning to the underlying neural connectivity patterns in Figure 9 (please see below).

More generally, the task is quite simple and the level of "generalization" (i.e. aim with the fixed offset) is fairly trivial, especially for participants that show explicit understanding. Why learning and transfer of such a task should involve apparently profound "reorganization" of connectivity, and how persistent such reorganization might be?

Response:
We thank the reviewer for these comments and for raising these points regarding the interpretation of our connectivity findings, particularly the link between behavior, explicit strategy, and our neural results. *Note that Figs. 8 and 9 referenced by the reviewer are now Figs. 9 and 10 in the revised manuscript, respectively (due to the inclusion of a new Fig. 6).

1. **Relating explicit learning (in new Fig. 9) to connectivity changes (in new Fig. 10):** We appreciate the reviewer highlighting the analyses in Fig. 9, which demonstrate significant inter-subject variability in task performance (angular error) and, critically, establish a direct link between performance and the use of explicit re-aiming strategies (Figs. 9D, E). That is, participants reporting a larger re-aimign strategy generally exhibited lower angular errors. Figure 10 then investigates the neural correlates of these individual differences by relating variability in task performance (angular error) to changes in network manifold eccentricity, which points to the involvement of the DMN-A network. The critical connection, which we have now clarified in the manuscript text, is that because lower angular error is strongly associated with better explicit strategy use (from Fig. 9), the correlation observed in Figure 10 between DMN-A eccentricity changes and lower angular error provides strong evidence for a relationship between DMN-A dynamics and the successful implementation of explicit strategies. We have revised the text introducing Figure 10 in the Results section to explicitly state this link, clarifying that the neural correlations with performance are likely reflect the engagement of explicit cognitive processes:

   "To test this, for each brain region and each of the RH early learning and LH early transfer epochs, we calculated the correlation between subjects' median angular error and the corresponding change in eccentricity from the Baseline to Early epochs for each hand (i.e., RH Learning Early > RH Baseline & LH Transfer Early > LH Baseline, Figs. 10A and D, respectively). [Note that for an analysis of the correlation between the eccentricity change from LH Baseline to LH Transfer Early versus transfer rate, please see Supplementary Fig. 6]. Crucially, because individual differences in angular error during these phases are strongly linked to the reported use of explicit strategies (as established in Fig. 9D, E), these brain-behavior correlations also allow us to probe, at least in part, the neural dynamics underlying the engagement of explicit cognitive processes during adaptation and transfer."

2. **Task simplicity vs. neural reorganization:** Regarding the apparent simplicity of the task versus the observed connectivity reorganization, it is worth recognizing that while the visuomotor rotation is a standard paradigm, the underlying cognitive and neural processes are demanding. Adaptation requires overriding prepotent actions, forming and implementing strategies, monitoring errors, and updating internal models, while generalization requires context evaluation and the retrieval of pre-established learning strategies. Our manifold analysis is specifically designed to be sensitive to the distributed, network-level dynamics supporting these processes. The observed 'reorganization' thus

reflects the brain actively engaging in these fairly complex adaptive mechanisms, rather than merely the task's superficial complexity.

3. **Persistence of reorganization:** Finally, the reviewer raises an excellent point about the persistence of these neural changes. Our analyses indicate that the distinct patterns of manifold reorganization, particularly involving the DMN, were most pronounced during the early phases of both learning and transfer (Fig. 5E). Furthermore, our new analysis directly examining the re-expression of the early learning pattern (in Fig. 6) demonstrates that the similarity of this specific network structure is highest between RH Early Learning and LH Early Transfer (as compared to the other learning phases). This suggests the DMN reorganization linked to initial adaptation and generalization is somewhat transient and phase-specific within our experimental timeframe (nevertheless, we acknowledge that the longer-term persistence of these connectivity patterns is an important area for future study).

2) It is functional connectivity study, using, at the core, inter-regional correlations (or covariance) matrices of ROI-based BOLD time series. The methods explain in some detail a denoising pipeline using confound regressors using pre-packaged preprocessing pipeline. But what about the task-related regressors? The typical application of such functional connectivity approaches mainly focuses on resting-state data, where there is no task-specific variance. There are several methods for calculating functional or effective connectivity in task-related data, but to the best of my understanding, they all include, in one way or another, task regressors to account for activation-induced changes in connectivity.

Response:
The reviewer raises a pertinent methodological point regarding our use of covariance-based functional connectivity in a task-based fMRI study, particularly given the existence of more explicitly task-modulated connectivity analysis techniques such as Psychophyiological Interaction (PPI) analysis, Dynamic Causal Modelling (DCM), and Univariate GLM-based approaches. While these methods offer valuable tools for examining task-related neural interactions, we contend that our chosen approach, focusing on covariance-based functional connectivity and manifold learning, is not only valid for task-based data but also offers unique advantages for addressing our specific research question concerning the large-scale network reorganization underlying motor generalization.

Firstly, it is important to clarify that covariance-based functional connectivity analysis is increasingly recognized and utilized as a valuable tool in task-based fMRI research, extending beyond solely resting-state applications (e.g., Gratton et al., 2018; Bassett & Mattar, 2017; Bassett et al., 2014; Nick et al., 2024; Rowchan et al., 2025; Zhu et al., 2025). This approach, by capturing the statistical dependencies between regional BOLD signals across time, provides a robust measure of inter-regional communication and network organization during task performance. Our study leverages this strength to examine how the overall architecture of brain-wide functional connectivity, as captured by manifold embedding, dynamically shifts across different phases of motor learning and transfer. This macroscale, whole-brain perspective, focusing on the emergent properties of network organization, is a key strength of our approach.

Secondly, while methods like PPI, DCM, and GLM-based connectivity offer the advantage of explicitly modeling task-related modulation of specific connections, they also come with key limitations that make our chosen approach particularly well-suited for our research objectives. Psychophysiological Interaction (PPI) analysis, for instance, is primarily focused on hypothesis-driven, pairwise interactions between pre-defined ROIs, examining how the relationship between two regions is modulated by task context. In contrast, our manifold analysis is inherently whole-brain and data-driven, allowing us to explore emergent patterns of network reorganization across the entire cortex, subcortex, and cerebellum without *a priori* assumptions about specific region-to-region interactions. Similarly, Dynamic Causal Modeling (DCM), while powerful for inferring effective connectivity and testing specific network models, requires strong *a priori* assumptions about network architecture and driving inputs, and can be computationally demanding for whole-brain analyses. Our covariance-based FC and manifold approach, being less reliant on strong prior assumptions, allows for the data-driven discovery of large-scale network dynamics.

Finally, while GLM-based connectivity approaches (e.g., beta-series correlation) can link task-evoked activity to connectivity, they often remain rooted in the analysis of univariate activation patterns. Our covariance-based FC, conversely, directly examines the multivariate covariance structure of the BOLD signal, focusing on network-level dependencies that may be independent of, or go beyond, simple task-evoked activation changes. Indeed, as demonstrated in our Supplementary Figure 4, our connectivity findings are not simply driven by changes in regional activation levels.

In summary, while we acknowledge the value of task-modulated connectivity methods like PPI, DCM, and GLM-based FC for addressing specific hypotheses about task-related neural interactions, we believe that our covariance-based functional connectivity approach, combined with manifold learning, offers a uniquely advantageous and complementary perspective for investigating the large-scale, data-driven reorganization of whole-brain network architecture that underlies sensorimotor learning and generalization. Of course, future research could certainly benefit from integrating task-modulated connectivity methods with manifold analysis to provide an even more comprehensive understanding of these complex dynamic processes, but these would be well beyond the scope of our present paper.

Nevertheless, given the reviewer's interest in these questions (and expecting that other readers may have similar questions), we have now added a new paragraph to the Methods section motivating our specific approach:

"**Manifold Construction.** Following the estimation and centering of epoch-specific covariance matrices, our subsequent analyses utilized manifold learning techniques to characterize large-scale network reorganization[12,17,47,68]. This analysis pipeline, focusing on the whole-brain functional connectivity structure captured in covariance matrices derived from task-based BOLD signals [21,23–25,30,103,164–167], was specifically chosen for its ability to provide a data-driven, whole-brain perspective on distributed network dynamics underlying learning and generalization. By embedding the connectivity profile of all brain regions into a low-dimensional space, this approach allows for the investigation of emergent macroscale shifts in functional architecture without the prerequisite of defining specific seed regions or pre-specifying network models, offering insights complementary to methods primarily focused on univariate activation or specific

task-modulated pairwise interactions (e.g., Psychophysiological Interaction (PPI), Dynamic Causal Modeling (DCM)[168,169]). The procedures for manifold construction based on these centered covariance matrices are detailed below."

3) My main concern is that the current presentation of results is hard to follow, especially for a non-expert in advanced dimensionality reduction methods. In particular, the meaning of manifold eccentricity, on which many results are based, need to be clarified and explained in a more consistent manner.

To start, the sentence in the abstract, "Here we show, using human functional MRI and a sensorimotor adaptation task involving the transfer of learning from the trained to untrained hand, that the transfer phase of adaptation reinstantiates the same cortical manifold structure as observed during initial adaptation." Perhaps I am underestimating the audience but I think "cortical manifold structure" would be difficult to understand for most people, and indeed, some might interpret it in a completely different way to what is intended by the authors.

Response:
We thank the reviewer for highlighting the potential lack of accessibility of the term 'cortical manifold structure' in the Abstract. We agree that more direct language is appropriate for a broad audience. We have revised the abstract to focus on the underlying neural phenomenon in more accessible terms. We include the newly revised abstract below:

“An often-desired feature of motor learning is that it generalizes to untrained scenarios. Yet, how this is supported by brain activity remains poorly understood. Here we show, using human functional MRI and a sensorimotor adaptation task involving the transfer of learning from the trained to untrained hand, that the transfer phase of adaptation re-instantiates a highly similar large-scale pattern of brain activity to that observed during initial adaptation. Notably, we find that these neural changes, rather than occurring at the level of sensorimotor regions, predominantly occur across distributed areas of higher-order transmodal cortex, specifically in regions of the default mode network (DMN). Moreover, we show that these learning-related neural changes relate to the structural properties of transmodal cortex (its myelin content and neurotransmitter receptor density), and that intersubject differences in DMN activity relate to both adaptation- and transfer-phase task performance. Together, these findings suggest that the transfer of learning across the hands is supported by the re-expression of the same activity patterns in the DMN as those that support initial learning. Collectively, these results offer a unique characterization of the whole-brain macroscale changes associated with sensorimotor learning and generalization, and establish a key role for higher-order brain areas, such as the DMN, in the transfer of learning to untrained scenarios.”

To continue with the Results, Figure 1C, what do the surface map represent, and how are they constructed from centered covariance matrices? There is no explanation neither in text nor in the legend, and the color bar with "arbitrary units" does not help. It took me some time, after reading further and back, to realize that these maps probably represent the first PC of the corresponding epoch, although I could not match it to Figure 2 - are PC1s shown in Figure 2 representing the average of left and right hand PC1 from Figure 1?

Response:
We thank the reviewer for pointing out the lack of clarity regarding Figure 1C; we apologize for this oversight and appreciate the opportunity to improve the explanation.

The reviewer is correct in deducing that the surface maps in Figure 1C visualize the first Principal Component (PC1) derived from the functional connectivity data. Specifically, each map represents the spatial pattern of PC1 loadings for a representative subject calculated from their corresponding covariance matrix for that particular task epoch (LH Baseline, RH Baseline, RH Early Learning, etc.). PC1 captures the dominant axis of variation in whole-brain functional connectivity for that specific epoch, essentially representing the primary dimension of that epoch's average connectivity manifold.

The color bar, which was labeled "arbitrary units," actually represents the PC1 loading value for each brain region, indicating how strongly each region contributes to that epoch's dominant connectivity pattern. We have corrected the color bar label accordingly in the revised figure.

Crucially, the reviewer also asked about the relationship between the PC1 maps in Figure 1C and the PCs shown in Figure 2. This is an important distinction:

- Figure 1C: Shows the PC1 calculated separately for the connectivity matrix of the representative subject for each individual task epoch. Its purpose is illustrative, showing how the dominant connectivity pattern differs across the raw epoch data before alignment to a common space.
- Figure 2: Shows the top three PCs (PC1, PC2, PC3) calculated from the single template Baseline manifold, which was derived by averaging the LH and RH Baseline connectivity data across *all* subjects. This Baseline manifold serves as the common reference space for all subsequent analyses, including the Procrustes alignment of individual subject/epoch manifolds and the calculation of manifold eccentricity.

Thus, while both figures show principal components, Figure 1C illustrates the changing primary pattern across different epochs for a single subject, whereas Figure 2 defines the stable reference frame derived from baseline conditions used for comparing all epochs.

To address this lack of clarity, we have:

1. Thoroughly revised the legend for Figure 1C to explicitly state that the maps show PC1 loadings from a representative subject for each epoch-specific connectivity matrix and explain what these loadings represent. In our Fig. 1 caption we now write the following:

   "**Figure 1: Task structure and overview of fMRI analysis.** (A) MRI setup (top) and task structure (bottom). (B) Average participant learning curves (mean across 8-trial bins). Orange and green traces denote periods during the task in which the left hand (LH) and right hand (RH) were used, respectively. Banding around each trace denotes +/- 1 standard error of the mean (SEM). Inset bar graphs at right compare the initial error (first 16 trials) for RH learning and LH transfer trials. ** denotes $p < 0.01$ (C) Neural analysis approach. *Right,* For each participant, we extracted timeseries data from the Schaefer 400 cortical parcellation, Tian 32-region subcortical parcellation and

Nettekoven 32-region cerebellar parcellation for six equal-length task epochs (indicated by the coloured boxes) and then computed functional connectivity (FC) matrices for each epoch. We then estimated connectivity manifolds for each task epoch using PCA with centered and thresholded connectivity matrices (see Methods). To visualize how the dominant pattern of FC changed across task phases before alignment to a common space, the surface brain maps depict the first Principal Component (PC1) loadings calculated from a representative subject's covariance matrices for each specific task epoch (e.g., LH Baseline, RH Baseline, etc.). The color bar indicates the PC1 loading value for each brain region, reflecting the strength and direction of that region's contribution to the epoch's primary connectivity pattern. These maps serve an illustrative purpose and will differ slightly from the first PC of the common template Baseline manifold shown in Fig. 2, which provides the reference space for all subsequent alignment and analyses. *Left,* Construction of this template Baseline manifold. All manifolds were aligned using Procrustes transform to a common template manifold created from a group-averaged FC matrix based on the mean across the LH and RH Baseline epochs. This allowed us to assess learning-related changes in manifold structure from this Baseline architecture. (D & E) Subject-level clustering is abolished through a Riemmanian centering approach. UMAP visualization of the similarity of FC matrices, both before centering (*D*) and after (*E*) centering. In these plots, each point represents a single FC matrix, color-coded either to subject identity (left panels) or task epoch (right panels), with its location in the two-dimensional space based on the similarity between matrices. Note that the uncentered connectivity matrices in A show a high-degree of subject-level clustering, thus obscuring any differences in task structure. By contrast, the Riemmanian manifold centering approach (in B) abolishes this subject-level clustering."

2. Corrected the color bar label to "PC1 loading".

3. Added a clarifying sentence to the main Results text when introducing Figure 1C, explaining what the maps depict and briefly noting their illustrative purpose prior to the main alignment analysis detailed later with Figure 2. We include this new sentence below:

   "Next, we applied dimensional reduction to the affinity matrices using principal component analysis (PCA) to obtain a set of principal components (PCs) that provides a low-dimensional representation (i.e., manifold) of cortical-subcortical-cerebellar connectivity structure for each epoch. The right panel in Fig. 1C shows the spatial pattern of the first PC (PC1) loadings derived from the centered FC matrix of a representative subject for each task epoch. Each brain region's color indicates the loading value (see Fig. 1C caption), illustrating shifts in the primary axis of connectivity organization across the task phases."

We believe these revisions make the content and purpose of Figure 1C, and its distinction from Figure 2, much clearer.

Perhaps more easily interpretable (i.e. imbued with functional meaning) headings would help, e.g. "Figure 2: Manifold structure and eccentricity during Baseline trials." can be replaced with "Figure 2: Main functional connectivity networks during Baseline trials"?

Response:
We thank the reviewer for this suggestion. To be even more exact, we have opted to title it: "Main connectivity axes extracted from baseline trials."

Along the same lines, Figure 3 is very dense and the associated text is hard to parse.

Response:
We thank the reviewer for this feedback regarding the density of Figure 3 and the clarity of the associated text. We acknowledge that this figure integrates several related analyses (UMAP visualization, empirical RSM, model RSMs, model comparison results) to provide a comprehensive view of the information encoded in the whole-brain eccentricity patterns across epochs, and we have revised the accompanying Results text to improve its structure and readability.

In our Results, we now write:

"***Global changes in manifold structure during learning and transfer***

We next sought to establish changes in manifold architecture across the different phases of the task. To this end, we first applied multivariate analytical methods to investigate, at the global whole-brain level, potential differences in manifold eccentricity across epochs. Fig. 3A visualizes the mean (across subject) whole-brain eccentricity for each of the six task epochs using two-dimensional UMAP [72]. In this two-dimensional projection, nearby points represent epochs with more similar whole-brain eccentricity patterns. This visualization suggests that multiple types of task-related information might be encoded in the global manifold structure...

...To complement these UMAP visualizations and quantitatively test specific hypotheses about the information represented, we performed Representational Similarity Analysis (RSA; [73,74]) on the eccentricity data. Using established methods [75] we constructed, for each subject and epoch, a representational similarity matrix (RSM) by calculating the Pearson correlation between the vectors of regional eccentricity values (464 regions) for every pair of the six task epochs (Fig. 3B shows the group-averaged RSM). This RSM captures the pairwise similarity between the neural states associated with each epoch based on their whole-brain eccentricity patterns.

We next tested how well this empirical RSM structure (Fig. 3B) was explained by three different *a priori* theoretical models representing distinct hypotheses about task structure (Figs. 3C, D): The first model predicted the encoding of hand information only ('Hand' model, Fig. 3C, left), the second predicted the encoding of the task epoch only (Baseline, Early or Late; 'Epoch' model, Fig. 3C, middle), and the third predicted the encoding of the Baseline versus Learning periods only ('Learning model'; Fig. 3C, right). These binary model RSMs represent theoretical similarity structures (where 1 denotes predicted similarity, 0 predicted dissimilarity) corresponding to each hypothesis. We compared each participant's empirical RSM to these theoretical model RSMs using cosine similarity, with higher values indicating a better fit between the model and the eccentricity data. The group-averaged results are shown in Fig. 3E. Statistical inference via bootstrapping revealed that both the 'Hand' and the 'Learning' model

performed better than the ‘Epoch’ model, with the ‘Learning’ model performing best (though not statistically better than the Hand model; see Fig. 3E).”

In addition, we have updated the Fig. 3 caption to better explain the different RSM models:

“**Figure 3: Multiplexing of task-based information in global manifold architecture.** (A) UMAP visualization of the similarity in neural states across task epochs (based on the data in B). In this plot, each point represents the across-subject mean whole-brain eccentricity for different task epochs (see legend at bottom), with nearby points representing more similar patterns of whole-brain eccentricity. Note that the two UMAP dimensions appear to capture different types of information about the task structure (see text). (B) Average across-subject representational similarity matrix (RSM) for the patterns of whole-brain eccentricity associated with the six task epochs (the value in each cell represents the Pearson spatial correlation). Note that the RSM is symmetric about the diagonal (black squares, which represent self-correlations). (C) Idealized model RSMs representing different hypotheses about task-related structure. (D) An idealized model RSM representing a control hypothesis based on the passage of time. In these model RSMs (C and D), values of 1 indicate predicted similarity between epochs according to the model, while 0 indicates predicted dissimilarity. These models are compared to the empirical data RSM shown in B. (E) Model comparisons. Each bar indicates the across-subject mean of cosine similarity between the RSM of the whole-brain eccentricity data (in B) and each of the models (in C and D; higher values mean higher similarity). Inference was performed via bootstrap resampling of subjects (1,000 bootstrap samples). Error bars indicate the standard error of the mean. Significance of each model for a one-sided comparison against zero is marked by white dew drops on the horizontal axis, and against the lower-bound estimate of the noise ceiling (gray icicles; FDR-corrected for 4 models). Models’ Pairwise differences are summarized by stars. ** denotes $q<0.05$.”

We believe these revisions to the text accompanying Figure 3 and the Figure 3 caption itself will provide a clearer step-by-step guide for the reader, making the figure's components and the logic of the analysis easier to parse despite the inherent information density.

The functional meaning of manifold eccentricity is hard to grasp, especially when considering one condition (e.g. baseline trials as in Figure 2). For instance, what does high eccentricity ("greater functional segregation") of visual cortex represent, in terms of underlying neuronal / structural connectivity? Does it imply that there is little connectivity between the visual cortex "network" and the rest of the brain? Similarly, what does the contralateral tuning of eccentricity mean, especially for the baseline periods (Figure 4)? Stronger eccentricity - stronger segregation? In other words, sensorimotor regions in the left hemisphere are less connected to the rest of the brain when using right hand (please see below)? How are the obvious consequences of the region-specific BOLD activity increase associated with contralateral hand movements factored in? Or are these activity changes subsumed under the manifold eccentricity?

Response:
The reviewer raises important questions regarding the functional interpretation of manifold eccentricity, and we appreciate the opportunity to clarify this concept, which can indeed seem abstract initially.

Manifold eccentricity, defined as the Euclidean distance of a region's position from the manifold centroid (coordinates [0,0,0] in our PCA space), serves as a multivariate index of that region's functional embedding within the whole-brain network architecture. It quantifies how unique or specialized a region's overall functional connectivity profile (its pattern of covariance with all other regions) is relative to the 'average' connectivity profile represented by the manifold's center. Regions with highly distinct connectivity patterns, often reflecting strong specialization within a specific functional system, tend to lie further from this average, resulting in higher eccentricity. Conversely, regions whose connectivity patterns are shared more broadly across different systems tend to lie closer to the center, exhibiting lower eccentricity. Thus, in network neuroscience terms, higher eccentricity generally indicates greater functional segregation (stronger coupling within its primary network and relatively weaker coupling with different networks), while lower eccentricity indicates greater functional integration (relatively stronger or more diverse coupling across different networks) [63–65]. Crucially, higher eccentricity does not imply weaker overall connectivity or that a region is 'disconnected' from the rest of the brain.

To empirically validate this interpretation above within our own data, we directly compared our baseline manifold eccentricity measure with classic graph theoretical metrics of network topology, calculated from the same underlying connectivity data (see Supplementary Fig. 2). This analysis confirmed that higher eccentricity significantly correlates with higher node strength (stronger overall connectivity) and higher within-module degree (stronger within-network connectivity), but lower participation coefficient (less diverse between-network connectivity). These correlations provide strong empirical support for interpreting higher eccentricity as reflecting greater functional segregation within the context of the whole-brain network.

Applying this interpretation, the reviewer's example of high eccentricity in visual cortex during Baseline trials (Fig. 2E) reflects its highly specialized function. Its connectivity pattern is distinct and strongly cohesive within the visual system, making it functionally segregated from the average brain region. Similarly, the contralateral tuning of eccentricity in sensorimotor cortex (Fig. 4) means that, for instance, left M1 exhibits increased connectivity with other sensorimotor regions when controlling the right hand compared to the left. This does not mean it is "less connected" overall when controlling the contralateral hand; rather, its connectivity pattern becomes more specialized and distinct relative to the whole-brain average during contralateral control (as explored further in our seed connectivity analyses, Fig. 8).

Finally, the reviewer asked how task-related BOLD activity increases are factored in. As detailed in our response to Reviewer #2 and shown in Supplementary Fig. 4, we performed control analyses demonstrating that the observed changes in manifold eccentricity are not driven by simple changes in mean BOLD activation levels across epochs. Thus, the eccentricity metric reflects shifts in the covariance structure of the BOLD signal, not merely its amplitude.

Given these comments above, we have added new text to our Methods to reinforce these points. In the Methods we now write the following:

"**Manifold Eccentricity.** Recent work has quantified the embedding of brain regions in low-dimensional connectivity spaces using Euclidean distance [21–23,69,70,170]. Here, we calculated "Manifold Eccentricity" as the Euclidean distance between a single region's coordinates in the aligned 3D manifold space and the manifold centroid (origin: [0,0,0]) [22]. This provides a

scalar index quantifying how typical or atypical a region's whole-brain functional connectivity profile is relative to the average profile represented by the centroid. Regions with highly distinct connectivity patterns (often reflecting functional specialization) tend to lie further from the centroid, resulting in higher eccentricity, while regions sharing connectivity features across multiple systems lie closer, yielding lower eccentricity. Thus, in network neuroscience terms, higher eccentricity generally indicates greater functional segregation (a more distinct profile, often with strong within-system coupling), while lower eccentricity suggests greater functional integration (a profile more similar to the average, reflecting broader cross-system coupling) [22,69,70]. Importantly, eccentricity reflects the relative distinctiveness of a connectivity pattern within the manifold, not simply overall connectivity strength or magnitude. To empirically validate this interpretation within our data, we confirmed that baseline eccentricity significantly correlated with established graph theoretical measures of network topology (see Supplementary Fig. 2), supporting its use as an index of functional segregation versus integration. These measures were calculated on the row-wise thresholded template connectivity matrix and included node strength, which is the sum of a region's connectivity weights; within-module degree z-score, which measures the degree centrality of a region within its respective network; and participation coefficient, which measures the network diversity of a region's connectivity distribution [171]. Cortical regions were assigned to their respective intrinsic functional networks [57,71] for these different graph theoretic calculations. For our main analyses, we computed the manifold eccentricity for each brain region, participant and epoch. This allowed us to statistically test for manifold expansions (increases in eccentricity) and contractions (decreases in eccentricity) throughout early and late learning and transfer trials. See our previous work [21,23] for a description of similar approaches."

We hope this detailed explanation above, and the changes to the text, clarifies the functional meaning of manifold eccentricity and its interpretation within our study.

What does the preferential tuning of the eccentricity for the left hand in both hemispheres in precuneus(?) might indicate (posterior blue region in Figure 4E)?

Response:
We thank the reviewer for pointing out this specific effect in the posterior cingulate/precuneus region (blue areas in Fig. 4E), where eccentricity appears preferentially tuned (higher) during use of the non-dominant left hand compared to the right hand. Interpreting this specific regional effect is less straightforward than the clear contralateral effects observed in sensorimotor cortex. However, we can offer a plausible, though speculative, interpretation. Given that the precuneus/posterior cingulate cortex is a core node of the DMN often implicated in monitoring and self-referential thought, it is conceivable that this higher eccentricity (i.e., higher within DMN connectivity) during left-hand trials reflects the potentially greater cognitive demands associated with actions performed by the less-practiced, non-dominant hand (in our right-handed sample). Such movements might necessitate heightened conscious monitoring, or the recruitment of cognitive control resources, even during baseline performance.

Nevertheless, given the speculative nature of this interpretation and the fact that it pertains to a specific modulation within a single higher-order region (contrasting with the broader network-level effects central to our main conclusions), we believe it is appropriate not to over-interpret its functional significance within the main manuscript. Our primary focus remains on the more robust and generalizable network-level findings concerning both Hand and Task Epoch effects.

Again, perhaps I am confused about the proper interpretation of manifold eccentricity changes, but the analysis in Figure 7 seems to contradict the interpretation of increased segregation: "Together, these results suggest that the manifold expansions of left and right M1 when using the contralateral hand mainly arise from their increased connectivity with other sensory-motor areas in cortex." It might be that the increased segregation "from other brain networks" can still be associated with increased connectivity a given "network", although the relative contribution of "within" vs "between" changes of connectivity still eludes me.

Response:
We appreciate the opportunity to further clarify this. As we detailed in our response to your earlier comment concerning the functional meaning of eccentricity, 'increased segregation' in the manifold context is a relative measure of a region's increased within-network connectivity (and does not equate to absolute disconnection or reduced overall connectivity). In the specific case of M1 in Figure 8 (note that Fig. 7 has been updated to Fig. 8 in the revised paper), the observed manifold expansion with contralateral hand use arises because its connectivity with other sensory-motor areas demonstrably increases. That is, the increase in left M1's connectivity with adjacent sensorimotor areas is greater when using the right hand than the left hand, and vice versa for right M1's connectivity. This connectivity effect is what drives the contralateral eccentricity effects.

To better emphasize this point to the reader, we have added the following new text to the Results section corresponding to Figure 8:

"Together, these results suggest that, generally speaking, the manifold expansions of left and right M1 when using the contralateral hand mainly arise from their increased connectivity with other sensory-motor areas in cortex. This highlights how increased connectivity within a functional system can lead to greater functional segregation of that system relative to the whole-brain average, manifesting as increased manifold eccentricity."

The analysis in Figure 9 first states that "Specifically, for the DMN-A networks (which mainly encompass mPFC, PCC and angular gyrus), greater expansion along the manifold during early learning corresponded to lower error (i.e., improved performance).", but then concludes that "Collectively, these findings suggest that greater integration of the DMN-A network with several other higher-order cognitive networks generally corresponded to a more effective reduction of subjects' visuomotor errors." I admit that I am not following those seemingly contradictory statements, since the expansion is presumably increase in eccentricity, which has been interpreted as increase in segregation through the manuscript?

Response:
The reviewer raises a crucial point regarding the interpretation of our findings for the DMN-A network (Figure 10) — specifically the relationship between its manifold expansion (increased eccentricity) correlating with better performance (lower error), and the seemingly contrasting finding that its integration with other cognitive networks also relates to better performance (lower error). We appreciate the opportunity to clarify this relationship, which we acknowledge was not effectively emphasized in our original text and hinges on considering the simultaneous changes in

DMN-A's connectivity with different systems and their relative positions on the whole-brain manifold.

As the reviewer notes, our results show that for the DMN-A network, a greater increase in manifold eccentricity (Early vs. Baseline) correlated with lower angular error (better performance) (Fig. 10B). Increased eccentricity signifies a greater functional segregation of DMN-A relative to the whole-brain average profile. To understand the specific connectivity changes driving both this increased eccentricity and the better performance, we examined the correlations between performance and the change in DMN-A's functional connectivity with all other systems (Fig. 10C). This analysis revealed a dual pattern associated with better performance:

1. Better performance (lower error) was associated with ***stronger*** functional connectivity between DMN-A and certain other higher-order cognitive networks (e.g., Control-B, SalVentAttn-B).
2. Better performance (lower error) was also associated with ***weaker*** functional connectivity between DMN-A and primary sensory and motor networks (e.g., visual, somatomotor).

These findings are in fact complementary and explain the eccentricity result when considering the geometry of the manifold (visualized in Fig. 2C). Strengthening connections with networks that are already similarly positioned along the manifold (like other cognitive networks near the transmodal pole) while simultaneously weakening connections with networks at opposing ends (like visual and somatomotor networks) will both contribute to increasing DMN-A's segregation and thus its eccentricity.

We now realize that, in our original text, we did a poor job of emphasizing this distinction which is really important to the interpretation. To address this, we have significantly revised the text in the Results section discussing Figure 10C to emphasize both aspects of this connectivity pattern (i.e., increased integration with cognitive networks located nearby on the manifold, decreased integration with sensory/motor networks located distantly) and clarify how this explains the link between increased eccentricity and improved performance. We now write the following:

"For the bilateral DMN-A networks, Fig. 10C shows that *increased* functional connectivity with the Control-B and SalVentAttn-B networks tended to be associated with lower visuomotor errors (i.e., better performance; these negative correlation relationships are denoted by blue colors on the brain maps). Conversely, we also found that better (worse) performance tended to be associated with *decreased* (increased) functional connectivity between the DMN-A and sensory and motor areas, as well as motor areas of the cerebellum (denoted by red colors in the brain maps)(see Supplementary Fig. 7 for interpretations of the other network effects). The concurrent relative strengthening of DMN-A connectivity with cognitive networks (e.g., Control-B and SalVentAttn-B), which are located at similar positions along the whole-brain manifold (Fig. 2C), versus relative weakening of DMN-A connectivity with sensorimotor networks (visual, somatomotor regions), which are located at opposing ends of the whole-brain manifold (Fig. 2C), likely explains our observed eccentricity result — namely, the relative increase in the DMN-A's eccentricity during early learning (see also Fig. 8 C,D). Moreover, our brain-behaviour correlation findings further suggest that the relative magnitude of this concurrent strengthening/weakening functional connectivity pattern relates to subject performance."

All in all, I think the meaning if eccentricity, supposed to serve as a "multivariate index" of connectivity, needs to be unpacked and clarified better, and perhaps the limitations of using a single number measure encompassing and averaging over enormous diversity of potential underlying effects should be discussed.

Response:
We agree with the reviewer that while manifold eccentricity provides a valuable summary, it's important to acknowledge its limitations as a single index derived from complex connectivity patterns. We appreciate the suggestion to discuss this explicitly.

As the reviewer notes, manifold eccentricity, by design, simplifies intricate connectivity changes into a single scalar value. Its strength lies in providing a statistically tractable multivariate index of a region's overall functional segregation or integration within the whole-brain manifold, enabling comparisons at a macroscale level. However, this simplification inherently involves averaging over diverse underlying effects and entails a loss of granular detail regarding specific connectivity changes. To address this limitation and provide a more detailed view, our study employed complementary seed-based connectivity analyses to unpack the specific patterns of inter-regional covariance changes contributing to the observed eccentricity effects (see Figure 8 for example seed regions, and Figure 10 for network-level connectivity correlations related to performance). We believe this combined approach effectively balances the need for a robust, whole-brain overview (via eccentricity) with the exploration of finer-grained connectivity details (via seed/network analyses).

To ensure this point is clear to readers, we have now explicitly discussed the inherent limitations of manifold eccentricity as a neural measure, alongside its strengths and our use of complementary analyses, in a new paragraph added to the Discussion section (in the Methodological Consideratios section):

"Finally, while manifold eccentricity offers a valuable and statistically tractable approach for characterizing the functional embedding of brain regions within a low-dimensional manifold space, it is important to acknowledge its inherent limitations as a single, summary measure. By design, eccentricity reduces the complex, multivariate patterns of regional connectivity to a single scalar value, inevitably averaging across a diverse array of underlying connectivity effects. This simplification, while facilitating group-level comparisons, necessarily entails a loss of information regarding the full richness and heterogeneity of dynamic network interactions. To mitigate this inherent limitation, our study also employed a complementary seed-based connectivity analysis to unpack the more granular patterns of inter-regional covariance changes that contribute to the observed manifold eccentricity effects. Thus, while manifold eccentricity provides a powerful lens for examining macroscale shifts in functional segregation and integration, its interpretation should be considered in the context of these complementary, more fine-grained analyses."

4) The valid point is made that only very few prior studies investigated similar paradigms with fMRI and they focused on more standard activation amplitude analysis within individual brain regions (but see Tzvi et al 2020, link below), while the present study goes beyond this work by looking at global whole-brain connectivity. But why there was no attempt to bridge the two types of analyses directly, in the same subjects, by analyzing also task-related activity using

commonly used GLM approaches? I think this comparative approach would greatly enhance the scope and the interpretability of this work, and will help addressing the point (3) above.

Response:
The reviewer raises an interesting point regarding the potential value of integrating our connectivity findings with more traditional GLM-based activation analyses. Recognizing that these approaches capture fundamentally different aspects of neural activity — GLMs measuring mean activation levels *within* regions, and our primary analysis measuring covariance *between* regions — we would indeed anticipate distinct rather than converging results. Nonetheless, we agree that comparing these perspectives could enhance the scope of our work and address potential questions about activation correlates.

To address this, we have now performed a standard mass-univariate GLM analysis using the same task epochs defined for our connectivity analyses. The methods for this analysis are detailed in the revised Methods section ("General Linear Model (GLM) Analyses"), and the results for key contrasts related to learning and transfer are presented in a new supplementary figure (Supplementary Figure 5).

As anticipated, and reflecting the different neural phenomena measured, the spatial patterns revealed by the GLM contrasts differ considerably from those highlighted by our primary manifold eccentricity analyses. For instance, while the GLM revealed specific activation changes during early learning primarily in parietal, visual, and inferior frontal gyrus regions (see Supplementary Figure 5B, below), partly consistent with prior activation-based studies of visuomotor adaptation (Anguera et al. 2007; Anguera et al. 2010; Seidler and Noll 2008), these localized patterns do not align with the large-scale, DMN-centric connectivity reorganization linked to learning and transfer effects in our main analyses. Indeed, the effects revealed through this analysis are much sparser, and much less organized according to functional networks. We include the supplementary figure, and its associated caption, below:

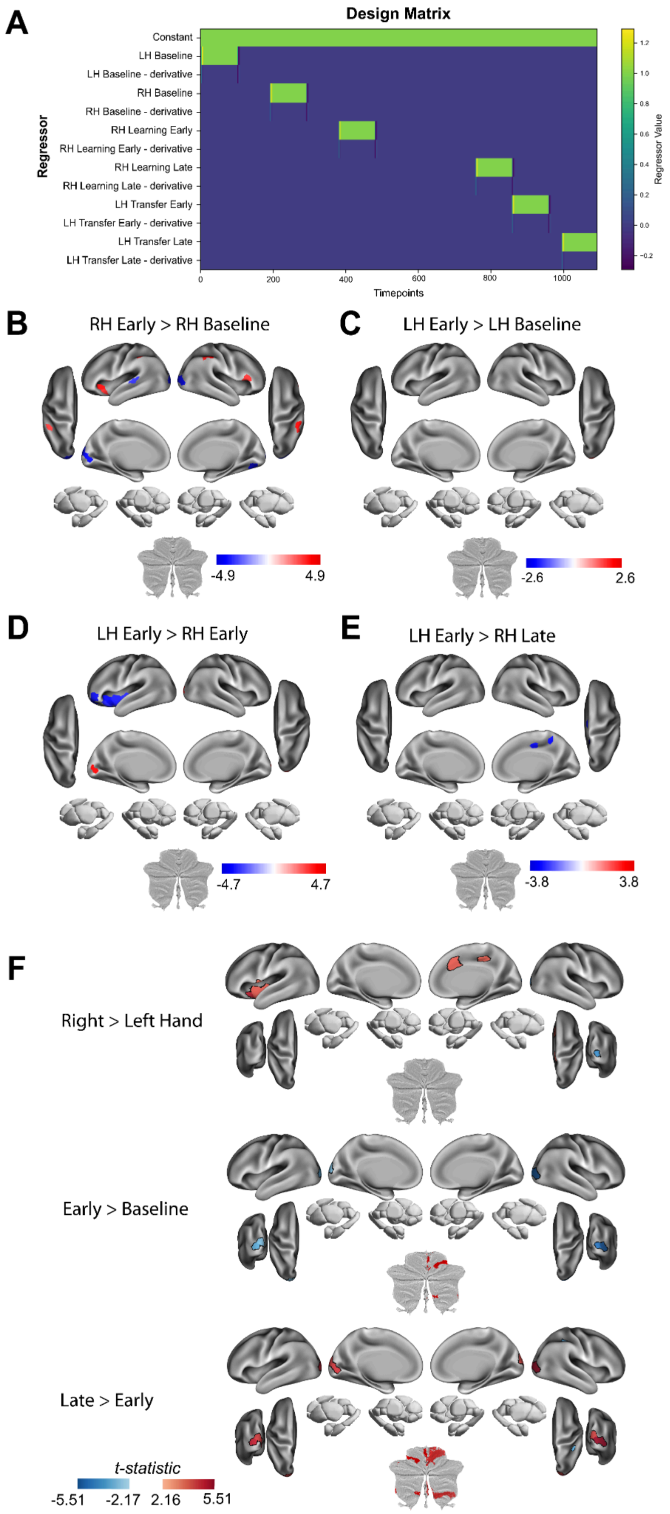

Supplemental Figure 5: Task-related modulations in regional BOLD activity revealed by GLM analysis. (A) Design matrix used for GLM modeling, convolved with the canonical Glover hemodynamic response function and its temporal derivative. Each regressor corresponds to one of six task epochs spanning baseline, learning, and transfer conditions. (B) Statistical map

for the contrast RH Learning Early > RH Baseline, revealing regions selectively engaged during early stages of right-hand learning. (C) Statistical map for the contrast LH Transfer Early > LH Baseline, capturing regions recruited during early transfer to the untrained hand. (D) Contrast LH Transfer Early > RH Learning Early, identifying regions more strongly engaged during transfer compared to initial learning. (E) Contrast LH Transfer Early > RH Learning Late, highlighting regions exhibiting greater activation during transfer relative to late learning stages. All maps display z-scores derived from group-level one-sample t-tests on subject-level beta estimates, thresholded at $q < 0.05$ using FDR correction. (F) To directly compare with the main functional connectivity analyses (Figs. 4 & 5), we performed an identical 3x2 repeated-measures ANOVA (rmANOVA) using the GLM beta estimates, with factors Task Epoch (Baseline, Early, Late) and Hand (Left, Right). This panel shows post-hoc contrasts for regions exhibiting significant main effects in this activation-based rmANOVA. Specifically, Right > Left contrasts are displayed for regions with a main effect of Hand, while Early > Baseline and Late > Early contrasts are shown for regions with a main effect of Task Epoch. This highlights condition-specific BOLD activity modulations analogous to the eccentricity changes examined in the main text.

We believe that including this GLM analysis in the supplement provides the requested comparison and confirms that our primary connectivity-based findings offer particularly unique insights into network-level reorganization that are complementary to, and not redundant with, standard activation mapping. To integrate this into the main text, we have added a brief mention of the GLM analysis in the Results section, directing the reader to the supplementary figure.

We now write the following:

"For completeness, and to provide a comparison with these functional connectivity-derived results, we also performed a standard mass-univariate General Linear Model (GLM) analysis to identify brain regions showing significant changes in mean BOLD activation across the task epochs (see Methods). The results of this analysis (Supplementary Fig. 5) revealed a distinct pattern of activation changes across epochs, which, while partly consistent with prior activation-based studies of visuomotor adaptation [8,78,79], did not closely correspond to the DMN-centric connectivity reorganization highlighted by our manifold analyses above. This divergence underscores that GLM activation and functional connectivity capture fundamentally different, albeit complementary aspects of neural activity underlying learning and transfer."

And, finally, in our Methods section, we provide the detailed methods for constructing our GLM-based analyses:

"**General Linear Model (GLM) Analyses.** To assess task-related changes in regional BOLD activity complementary to our primary connectivity analysis, we applied a standard mass-univariate general linear model. For this analysis, to ensure comparability, we used the same preprocessed, parcellated fMRI time series data spanning the cortex (Schaefer 400 cortical parcellation [172]), subcortex (Tian subcortical (scale II) 3T subcortical atlas [58]), and cerebellum (Nettekoven cerebellar atlas [59]). The design matrix for each participant included regressors for each of the six task epochs used in the connectivity analyses (LH Baseline, RH Baseline, RH Learning Early, RH Learning Late, LH Transfer Early, LH Transfer Late). Each epoch was modeled as a sustained block encompassing the relevant 32 trials (96 TRs) and convolved with the canonical Glover

hemodynamic response function (HRF) along with its first temporal derivative [173,174] to account for latency variations (12 regressors total). A constant term (intercept) representing the session mean was also included. Subject-level beta estimates for each condition were estimated using ordinary least squares regression, implemented via the nilearn run_glm function. Group-level statistical analyses were conducted using paired-samples t-tests on the beta estimates for several predefined contrasts of interest relevant to learning and transfer (e.g., RH Learning Early > RH Baseline, LH Transfer Early > LH Baseline, LH Transfer Early > RH Learning Early, and LH Transfer Early > RH Learning Late), as detailed in Supplementary Figure 5. Resulting group-level statistical maps (z-scores) were corrected for multiple comparisons across all 464 brain regions using the Benjamini-Hochberg false discovery rate (FDR) procedure ($q < 0.05$). To assess task-related modulations in regional BOLD activity, we conducted a 3 × 2 repeated-measures ANOVAs (rmANOVAs) on the GLM beta estimates for each region, with Task Epoch (Baseline, Early, and Late) and Hand (Left vs. Right) as within-subject factors. Multiple comparisons were corrected using false discovery rate (FDR) correction ($q < 0.05$) across all tests (464 regions × 3 effects: main effects of Epoch and Hand, and their interaction). For regions exhibiting significant main effects, we performed follow-up post-hoc paired t-tests to compare Right vs. Left hand (for Hand main effect), and Early vs. Baseline and Late vs. Early epochs (for Epoch main effect), identifying specific condition-driven changes in BOLD activation."

Minor issues/clarifications:
"The target appeared at one of eight randomized locations, separated by 45° increments (0, 45, 90, 135, 180, 225, 270, and 315°), in bins of eight trials."
- was there a learning dynamics within each "bin" of 8 trials with the same target (and each new target increased the error)? Or it was mainly gradual learning regardless of the target bin? What was the reason for 8-trial bins?

Response:
The reviewer asks about learning dynamics within the 8-trial target bins and the rationale for this binning structure. It seems there may be a slight misunderstanding, as the task design, which is standard in the visuomotor rotation literature (e.g., Taylor et al., 2014; Mazzoni & Krakauer, 2006), did not involve 8 consecutive trials to the *same* target. Instead, targets were presented pseudorandomly such that each of the eight locations appeared once within every 8-trial bin. The primary reason for this structure is experimental control, ensuring a balanced presentation of all target directions to promote generalizable adaptation across the entire workspace, rather than biasing learning towards any single direction. Consequently, learning dynamics were not specific to these bins. As evidenced by the gradual error reduction shown in our learning curves (Fig 1B), adaptation is a cumulative process unfolding across many trials and target directions, rather than showing distinct patterns strongly tied to the 8-trial structure itself.

I think references and a discussion of the results of two relevant studies by Kirby et al and especially Tzvi et al are missing:
https://pubmed.ncbi.nlm.nih.gov/31542802/
https://www.sciencedirect.com/science/article/pii/S1053811920306285

Response:
We thank the reviewer for suggesting these relevant studies. We have now incorporated citations to both Kirby et al. (2019, reference #103) and Tzvi et al. (2020, reference #115) within the introductory

paragraphs of our Discussion section, where we review previous approaches to studying the neural correlates of sensorimotor adaptation.

Specifically, we now write:

"Previous fMRI studies investigating the neural bases of motor generalization have predominantly focused on motor sequence learning, examining the transfer of learned finger movement sequences across the hands (for reviews, see [107–111]). In contrast, research on the neural bases supporting the generalization of sensorimotor adaptation, which involves the transfer of learned action elements rather than their sequence [27,112,113], has been relatively scarce. Of the limited studies available, they have primarily focused on characterizing learning-related increases versus decreases in the activity of individual brain regions, such as visual, parietal, motor, and premotor cortical areas, as well as the cerebellum and striatum [1,7,8,78,79,114]. For instance, fMRI activation studies have highlighted the differential recruitment of these areas depending on whether the dominant or non-dominant hand undergoes initial training [103]. Other work has employed connectivity analyses, such as using Dynamic Causal Modeling (DCM) to probe specific interactions like those between the cerebellum and premotor cortex during adaptation [115]. While valuable, these approaches often focus on specific regions or pre-defined circuits. Yet, contemporary perspectives [1,116] suggest that it is functional interactions across distributed, large-scale networks that underpin learning-related processes. "

We believe this integration appropriately acknowledges these studies within the context of prior work employing activation-based or specific circuit-based connectivity analyses, while maintaining the focus of our Discussion on the rationale and findings related to our large-scale network approach.

Page 4: "By disentangling effector-specific (right versus left hand) versus effector-independent changes in manifold structure, we found that it was predominantly the connectivity of DMN areas, and not sensorimotor regions, that were modulated across both the learning and transfer phases of the task." - grammar? (was / were)

Response:
We think 'were' is appropriate here, given that the 'areas' is plural. In any case, we agree that the grammar is a little awkward here. We have now revised the text accordingly:

"By disentangling effector-specific versus effector-independent changes in manifold structure, we found that the connectivity of default mode network (DMN) areas, rather than sensorimotor regions, was predominantly modulated across both learning and transfer phases."

Page 24: "While significant changes in the manifold embedding of individual brain regions indicate an alteration in those regions' patterns of whole-brain connectivity, it is not obvious the precise nature of these connectivity alterations." - grammar? (the precise nature of these connectivity alterations is not obvious)

Response:
We agree that our grammar here was also awkward. We have changed the text based on the reviewer's recommendations. We now write the following:

“While significant changes in the manifold embedding of individual brain regions indicate an alteration in those regions' patterns of whole-brain connectivity, the precise nature of these connectivity alterations is not obvious”

Page 34: "...and that inter-individual differences in the manifold embedding of DMN areas was related to both learning and generalization performance." - grammar? (was / were)

Response:
Yes, the reviewer is correct that the appropriate grammar is ‘were’ here. We have revised this sentence accordingly:

“...and that inter-individual differences in the manifold embedding of DMN areas were related to both learning and generalization performance.”

Lastly, will the entire preprocessing and code be made available? Given the elaborated and highly abstracted pipelines, I think it would be very good for reproducibility.

Response:
Yes, all the preprocessed data (in BIDS formatting) will be made available (on OpenNeuro), along with all the code used to generate the analyses and results!

Lastly, we’d like to thank you for your positive and constructive comments on the paper!
